# An Integrated Care Pathway for depression in adolescents: protocol for a Type 1 Hybrid Effectiveness-implementation, Non-randomized, Cluster Controlled Trial

**DOI:** 10.1186/s12888-023-05297-4

**Published:** 2024-03-08

**Authors:** Darren B. Courtney, Melanie Barwick, Bahar Amani, Andrea T. Greenblatt, Madison Aitken, Karolin R. Krause, Brendan F. Andrade, Kathryn Bennett, Kristin Cleverley, Amanda A. Uliaszek, Claire de Oliveira, Lisa D. Hawke, Jo Henderson, Wei Wang, Priya Watson, Amy Gajaria, Amanda S. Newton, Stephanie Ameis, Jacqueline Relihan, Matthew Prebeg, Sheng Chen, Peter Szatmari

**Affiliations:** 1 Centre for Addiction and Mental Health, Toronto, ON Canada; 2 University of Toronto, Toronto, ON Canada; 3 Cundill Centre for Child and Youth Depression, Toronto, ON Canada; 4 Hospital for Sick Children, Toronto, ON Canada; 5 Department of Health Research Methods, Evidence, and Impact (Formerly Clinical Epidemiology and Biostatistics), McMaster University, McMaster University Faculty of Health Sciences, Hamilton, ON Canada; 6 Department of Pediatrics, University of Alberta, Edmonton, AB Canada

**Keywords:** Adolescent, Depression, Integrated care pathway, Measurement-based care, Implementation

## Abstract

**Introduction:**

Our group developed an Integrated Care Pathway to facilitate the delivery of evidence-based care for adolescents experiencing depression called CARIBOU-2 (Care for Adolescents who Receive Information ‘Bout OUtcomes, 2^nd^ iteration). The core pathway components are assessment, psychoeducation, psychotherapy options, medication options, caregiver support, measurement-based care team reviews and graduation. We aim to test the clinical and implementation effectiveness of the CARIBOU-2 pathway relative to treatment-as-usual (TAU) in community mental health settings.

**Methods and analysis:**

We will use a Type 1 Hybrid Effectiveness-Implementation, Non-randomized Cluster Controlled Trial Design. Primary participants will be adolescents (planned *n* = 300, aged 13–18 years) with depressive symptoms, presenting to one of six community mental health agencies. All sites will begin in the TAU condition and transition to the CARIBOU-2 intervention after enrolling 25 adolescents. The primary clinical outcome is the rate of change of depressive symptoms from baseline to the 24-week endpoint using the Childhood Depression Rating Scale—Revised (CDRS-R). Generalized mixed effects modelling will be conducted to compare this outcome between intervention types. Our primary hypothesis is that there will be a greater rate of reduction in depressive symptoms in the group receiving the CARIBOU-2 intervention relative to TAU over 24 weeks as per the CDRS-R. Implementation outcomes will also be examined, including clinician fidelity to the pathway and its components, and cost-effectiveness.

**Ethics and dissemination:**

Research ethics board approvals have been obtained. Should our results support our hypotheses, systematic implementation of the CARIBOU-2 intervention in other community mental health agencies would be indicated.

**Supplementary Information:**

The online version contains supplementary material available at 10.1186/s12888-023-05297-4.

## Introduction

### Background

Depression in adolescence is prevalent [1], debilitating [2] and a potent risk factor for suicide [3]. In Canada, publicly-funded community mental health agencies provide the majority of child and youth mental health care. Our group conducted a province-wide survey of services for the treatment of depression in children (≤ 12 years old), adolescents (13–18 years old) and transitional-aged youth (18–25 years old) that showed evidence-based treatments are not consistently implemented in the community. The survey also highlighted heterogeneity in the treatments offered [4]. A gap between what is scientifically supported in mental health care and what is practiced in the real world presents a missed opportunity to optimize treatment for depression in adolescents.

As a step towards bridging the research-practice gap, our group developed an Integrated Care Pathway (ICP) for treating depression in adolescents based on high-quality treatment recommendations [5], collaborative development efforts (including input from youth with lived experience) [6] and successful pilot testing [7]. The pathway is called CARIBOU-2 (“Care for Adolescents who Received Information ‘Bout Outcomes,” 2^nd^ iteration). The aim of the pathway intervention is to improve depressive symptoms in adolescents presenting to care by facilitating the delivery of multifaceted, youth-centred, and evidence-based care in community mental health agencies.

**Fig. 1:**
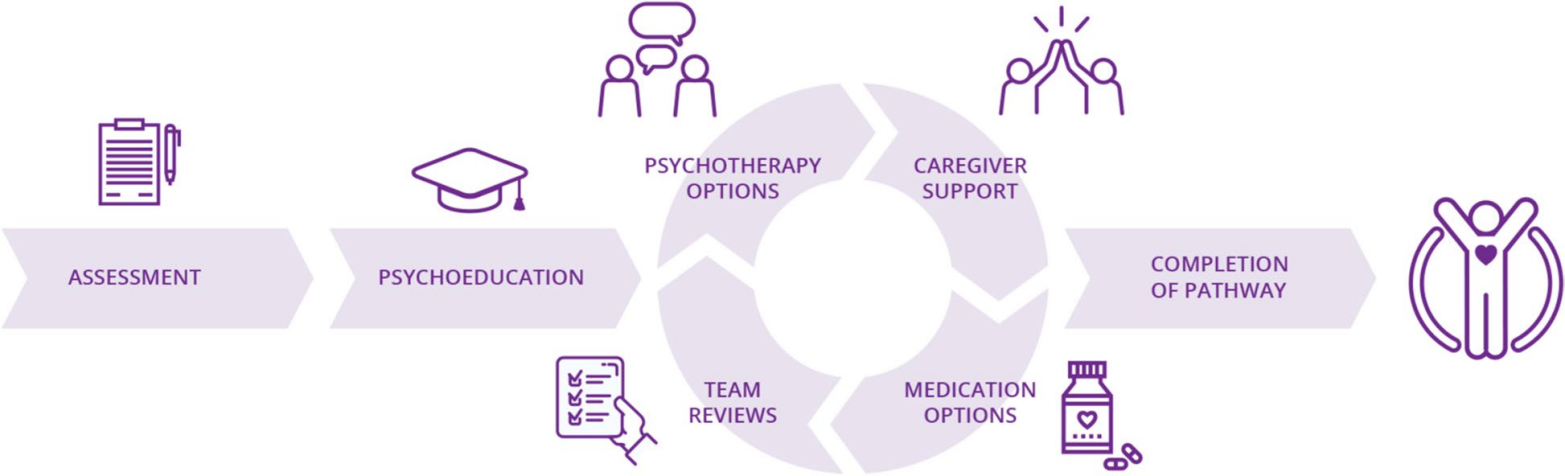
Schematic of the CARIBOU-2 intervention

The CARIBOU-2 intervention involves seven core components: (1) assessment; (2) a psychoeducation session; (3) psychotherapy options (1st line Cognitive Behavioural Therapy (CBT), 2nd line Brief Psychosocial Intervention [8] (BPI)); (4) a caregiver group; (5) medication options (1st line fluoxetine, 2nd line sertraline, 3rd line escitalopram, 4^th^ line duloxetine); (6) measurement-based care “team reviews” every four weeks (meeting with the adolescents and involved clinicians in reviewing measures and discussing treatment changes in a shared decision-making framework [9]); and, (7) graduation. Figure 1 outlines a schematic of these components. Development of the pathway and pilot study results are described elsewhere [6, 7, 10]. Documents and videos are available online describing the pathway in more detail [11].

### Theory of change

We propose that the CARIBOU-2 intervention will improve symptoms in adolescents with MDD-A through the following mechanisms:

1. *Enhanced implementation of evidence-based care: *Optimal evidence-based care synthesizes research findings, patient preferences, and clinician expertise with the finite resources of a given clinical setting [12, 13]. Evidence-based treatments are not consistently implemented for adolescents with depression in community mental health agencies [4, 14]. In the absence of intentional, explicit, and systematic implementation, uptake of evidence-based interventions in health care is slow, inefficient, and haphazard [15]. Implementation science provides the knowledge base to close this knowledge-to-practice gaps toward more effective and efficient health service delivery. This premise informed the development of the CARIBOU-2 pathway and will be used to guide implementation of the pathway components.
2. *Addressing complexity:* Single modality treatments for depression in adolescents (e.g., education, psychotherapy, medications or family work) often focus on a single system level that must be addressed (e.g., knowledge, or psychological processes, or biological mechanisms, or family relationships). To date, these focused treatments have had limited benefit [16, 17]. It is more likely that depressive symptoms are a function of complex interactions between these system levels [18]. Facilitating the delivery of coordinated, multifaceted care through the CARIBOU-2 pathway may address the complex nature of depression in adolescents, leading to improved outcomes.
3. *Measurement-based care:* There are no clear baseline moderators of outcome for depression in adolescents that can be applied at the individual level to preferentially recommend one evidence-based treatment over another as a starting point [19]. As such, including continual measurement of outcome throughout the course of the treatment is necessary to monitor progress and guide treatment adaptation decisions as needed. CARIBOU-2 achieves this level of monitoring through measurement-based care, the "the systematic administration of symptom rating scales that uses outcomes to drive clinical decision-making at the level of the individual patient” [20]. Research suggests that measurement-based care works by capturing treatment stagnation early and enabling the treatment team (including the person receiving the treatment) to correct course accordingly [20–22].
4. *Shared decision-making: *Clinicians delivering the CARIBOU-2 pathway will apply shared decision-making summarized using three principles [9]. Firstly, the decision involves the adolescent and clinician (a third person, such as a parent, may also be involved). Next, the decision involves exchanging important information with all parties; most often, the clinician provides information on treatment options, while the adolescent (and caregiver/parent) provide information on context, values, and goals. Lastly, all parties agree to next steps (note that the clinician or caregiver may not agree that it is the best option, but an acceptable one). Shared decision-making has been associated with improved health outcomes, though results are variable [23]. Some have posited that shared decision-making works to improve health outcomes by improving service user trust in the clinician, leading to greater adherence to the treatment [23].

Through a separate review [24], our group identified 98 randomized clinical trials of interventions for the treatment of depression in adolescents. Of these, only 4 studies tested the effectiveness of specific service delivery models and/or measurement-based care [25–28]. None of these studies tested the effectiveness and implementation outcomes of an ICP derived from high-quality guideline recommendations, nor did they extensively involve collaborative efforts with clinicians and youth in developing and implementing the intervention.

### Study objectives

The primary objective of this study is to test the clinical effectiveness of the CARIBOU-2 intervention delivered to adolescents with depression in community settings to reduce evaluator-rated depressive symptoms relative to treatment as usual (TAU). Our secondary objectives are to explore changes in self-rated and caregiver-rated depressive symptoms, and self-rated function (i.e., ability to adapt to demands of home, school, peers and community [29]) for adolescents receiving the CARIBOU-2 intervention relative to TAU. The third objective is to explore the implementation process and implementation effectiveness of CARIBOU-2 in the community settings including clinician fidelity to the intervention, cost-effectiveness, and acceptability of the intervention from the perspective of agency staff. Ultimately, this study aims to bridge the research-to-practice gap in community mental health agencies and optimize outcomes for adolescents with depression. TAU was chosen as the comparator as we ultimately want to answer the question of whether the CARIBOU-2 intervention should be recommended to replace current treatment practices in community settings [30].

### Hypotheses

I. Clinical Effectiveness Outcomes
*Hypothesis A (Primary)*: There will be a greater rate of reduction in blind evaluator-rated depressive symptoms in the group receiving the CARIBOU-2 intervention relative to TAU over 24 weeks as per the Childhood Depression Rating Scale-Revised (CDRS-R) [31].
*Hypothesis B*: There will be a greater rate of reduction in self-reported depressive symptoms in the group receiving the CARIBOU-2 intervention relative to TAU over 24 weeks as per the Mood and Feelings Questionnaire (MFQ) [32].
*Hypothesis C*: There will be a greater rate of improvement in self-reported functioning over a 24-week period as per the Child Anxiety and Depression Life Interference Scale-Youth Version (CADLIS-Y) [33] in the group receiving the CARIBOU-2 intervention, relative to TAU.
*Hypothesis D*: There will be a greater rate of reduction in caregiver-reported youth internalizing psychopathology symptoms over a 24-week period as per the Child Behaviour Checklist (CBCL) Internalizing Broadband subscale [34] in the group receiving the CARIBOU-2 intervention, relative to TAU.
II. Implementation Outcomes
*Hypothesis E*: The CARIBOU-2 intervention implementation process will be followed with ≥75% fidelity for each of the 6 sites as per a locally developed checklist.
*Hypothesis F*: The CARIBOU-2 intervention will be delivered with >75% fidelity to the overall ICP (i.e. how and when components are offered) as per a locally developed checklist.
*Hypothesis G*: The CARIBOU-2 intervention will be delivered with >75% fidelity for each separate psychotherapy component of the ICP (e.g., fidelity to Cognitive Behavioural Therapy) using established fidelity checklists where available, and locally developed checklists where no established checklist is available.
*Hypothesis H*: The CARIBOU-2 intervention will be cost-effective compared to TAU as determined through economic evaluation (protocol to be submitted for publication separately).

Acceptability of the CARIBOU-2 intervention to the agency staff, as well as implementation barriers and facilitators will be explored through qualitative methods with agency staff. Adolescent attendance at indicated sessions will also be reported. There are no associated hypotheses for these outcomes. 

## Methods and analysis

### Study design

An expanded version of the protocol, including the rationale for decisions made and regular updates, is available here: https://osf.io/6qzt7/. We used the relevant reporting guidelines to describe the protocol; namely, the Standard Protocol Items: Recommendations for Intervention Trials [35] and Standards for Reporting Implementation studies [36].

**Fig. 2:**
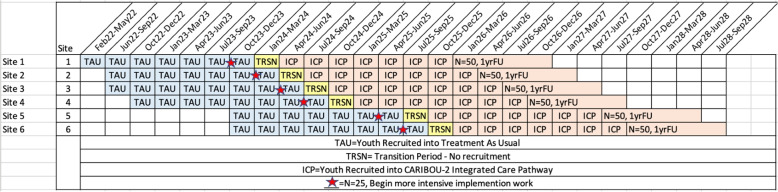
Allocation sequencing, recruitment of youth participants and follow-up at each site^a^ ^a^These are hypothesized timelines to reach *n* = 25 based on client volumes at each site, but the actual timeline may vary

The study is a superiority trial that uses a Type 1 Hybrid Implementation Effectiveness design [37] focusing on the clinical effectiveness of the CARIBOU-2 intervention while examining its implementation process and outcomes. The study design is a non-randomized, cluster controlled trial, graphically depicted in Fig. 2. The initial plan was to conduct a stepped-wedge design with a randomized sequence of newly implementing the pathway; however, we had to modify the design to account for differential rates of setup and recruitment across sites. The current design is pragmatic with no random participant or site allocation to treatment arm. Participating organizations will be six community mental health agencies, in Canada, where each site serves as the cluster unit. We are allocating treatment arm at the site level, and not the individual level, to minimize contamination effects; that is, once we train site staff in the pathway, this would potentially affect clinical outcomes in all adolescents at the site. In the first data collection phase all sites will remain in the Treatment as Usual (TAU) condition. Once a site has enrolled at least 25 adolescent participants into TAU, the transition from TAU to the ICP condition will begin. We intend a 1:1 ratio of adolescents allocated to each treatment arm within each site by the end of the trial. Implementation will be staggered with a minimum of 3 months between the onset of site transitions.

### Youth and caregiver engagement in research

We have collaborated extensively with youth partners (ages 13–25) through the development of the CARIBOU-2 pathway and related research. Prebeg and colleagues have detailed youth partner involvement in a preprint manuscript (2023) [38]. In parallel, a caregiver engagement coordinator will support caregiver advisors with relevant experience in the mental health system in providing relevant feedback to caregivers.

### Pathway implementation process

**Fig. 3:**
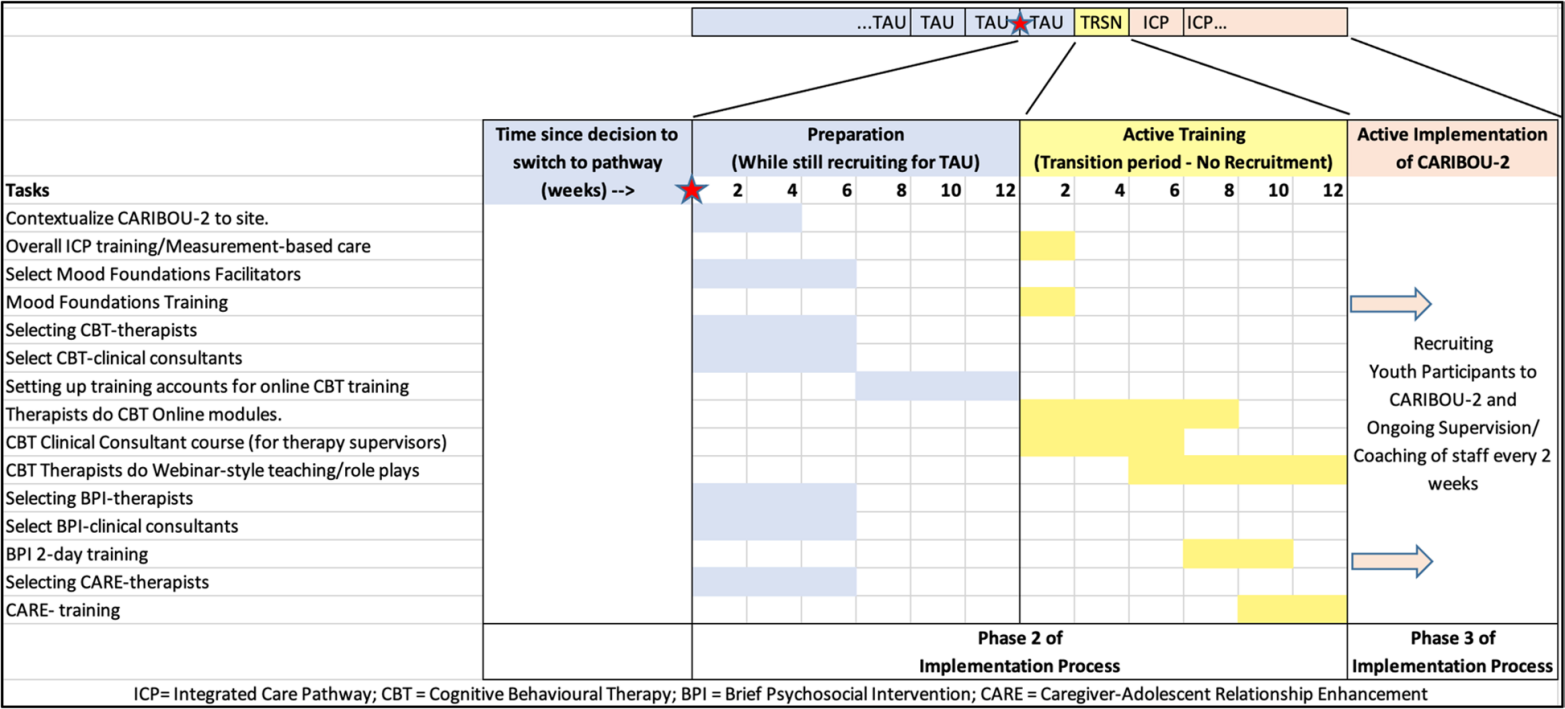
Timeline of steps to take place in phases 2 and 3 in implementation process

Implementation facilitation will be provided by members of the research team with relevant experience. The implementation process will be informed by the Quality Implementation Framework, which identifies four implementation phases and specific actions related to that will optimize attainment of quality implementation [39]. The four phases are as follows:

- * Phase 1- Initial considerations regarding the implementing organization:* Canadian community mental health agencies identified through networks associated with the research team were invited to a webinar wherein a detailed description of the CARIBOU-2 pathway and study details were discussed. Next, agencies that expressed interest in the study met with leads (DBC, MB, B Amani, ATG) to discuss implementation readiness in a separate virtual meeting informed by the Checklist to Assess Organizational Readiness [40] and the National Implementation Research Network Hexagon Tool [41]. Separately, each community mental health agency (“the site”) and the research team then collaboratively decided whether the respective site will be enrolled, up until all the first 4 sites were participating in the study. An additional 2 sites are being sought using similar methods.
- *Phase 2—Creating a structure for implementation:* Site-based, implementation teams will lead the planning and execution of CARIBOU-2 with supportive facilitation from members of the research team (MB, ATG). Implementation preparation during this phase includes ensuring sites have the capacity to deliver on all CARIBOU-2 core components and making requisite adaptations to existing clinical operations and resources where needed. Clinician training will occur at this time. See Fig. 3 below.
- *Phase 3- Ongoing support once implementation begins:* The key focus following the launch of the CARIBOU-2 intervention at each site will be on problem-solving barriers to delivery, providing coaching support to clinicians, and tracking fidelity and other outcomes. Ongoing support from the study leads (DBC, MB, B Amani, ATG) will be provided through: (i) biweekly clinical consultation between agency staff and the study leads (DBC, B Amani), and (ii) continuous process evaluation, which will involve site-specific implementation teams reviewing fidelity data to ensure that any changes to the model or approach are planned rather than reactionary. Site-specific implementation teams will meet with adolescent-facing clinicians every 2 weeks for 6 months to sustain the intervention through local supervision and a cross-site community of practice.
- *Phase 4- Improving future applications:* We will synthesize clinical and implementation outcome results and examine potential modifications to the pathway and implementation process that could inform future scale up.

### Participants

#### Primary participant recruitment (Adolescents)

Adolescent participant recruitment is taking place over 4.5 years from February 2022 to September 2027. Adolescents self-refer to the site or are referred by a third party (e.g., doctors, school counsellors, caregivers) and will be recruited to the study after their intake. Site staff (e.g., intake workers, clinicians) will conduct a screening assessment to confirm eligibility. The screening assessment, provided in Appendix A, is intended to mimic what would happen in typical clinical practice to identify adolescents eligible for the pathway. We do not require a diagnostic assessment, as this is not readily available at Canadian community mental health agencies.

The following inclusion criteria will be applied for adolescent participation:

- between 13 to 18 years and 11 months of age, inclusive;
- the adolescent and/or their caregiver express that ‘depression” (or some synonym) is a primary concern;
- a clinician or intake staff agree that depressive symptoms are a primary treatment target;
- a self-reported score of ≥ 22 on the Mood and Feelings Questionnaire (MFQ) [32], which represents clinically significant depression [42], at two sequential visits (screening and baseline assessment);
- either a new referral to the clinic within the past 3 months or, if previously treated at the clinic, has had a period of 3 months without treatment within the past 6 months;
- ability to speak and read English as per self-report and clinician impression.

As operationalized in Appendix A, adolescents will be excluded from the study if they have known or highly suspected:

- presentations of psychotic symptoms that are persistent, affect functioning, and have observable effects on behaviour;
- severe substance use disorder, bipolar disorder, intellectual disability, severe eating disorder, or imminent risk of suicide requiring hospitalization;
- an inability to provide informed consent to the study for any reason.

Youth partners co-designed one-page infographics about the study to enhance relevance and promote enrollment. These are provided to candidate adolescent participants by site staff to provide a summary of the study prior to the consent process. If the adolescent is eligible and agrees to be contacted by the research team, a consent meeting is arranged. Informed consent will be obtained from all study participants by a research assistant through a secure videoconference meeting. If the adolescent consents at enrollment, caregivers will be contacted to promote follow-up data collection. Site implementation leads (e.g., managers and/or senior clinicians) will meet monthly to monitor enrollment rates and innovate strategies to promote recruitment and retention of youth participants.

##### Secondary participant recruitment (Caregivers)

With adolescent assent, caregivers will be invited to participate in the study. A caregiver is any adult in a primary caregiving role for the adolescent (e.g., a parent).

##### Tertiary participant recruitment (Site operational implementation team members, supervisors and clinicians)

Supervisors and clinicians interested in participating will be recruited for the study. Clinicians must be social workers, social service workers, occupational therapists, nurses, psychologists, psychiatrists, or registered therapists to deliver the interventions. Learners supervised by these clinicians (e.g., social work student participating in a clinical rotation for their schooling) may also provide the intervention.

Inclusion criteria for Secondary and Tertiary participants:

- age 18 years and older;
- ability to read and write in English as per self-identification.

Exclusion criterion for Secondary and Tertiary participants:

- an inability to provide informed consent to the study for any reason.

### Interventions

#### Treatment as usual

TAU may or may not involve any of the following: assessment, education, various types of therapy, medications, and family work. There is no prescribed format to any of these interventions, nor prescribed measurement-based care. We have developed a clinician-reported checklist of common approaches to psychotherapy (e.g., CBT) that will be applied through chart review to characterize the treatment for each youth TAU participant (see Appendix B).

#### CARIBOU-2 Intervention

##### Core component 1. Initial youth and caregiver clinical assessment

As part of the CARIBOU-2 intervention, youth will undergo an assessment by a clinician at the site that includes various measures intended as a baseline for measurement-based care. These are measures of depression (Mood and Feelings Questionnaire—MFQ) [32], anxiety (Revised Children's Anxiety and Depression Scale-25– Anxiety Subscale (RCADS-anx-25)) [43], function (Child Anxiety and Depression Life Interference Scale-Youth version (CADLIS-Y) [33], Patient Global Impression (PGI) –Severity scale [44], and Goals Based Outcome (GBO) [45]. Youth indicating a risk of suicide (answering at least a “sometimes” on items 16–19 that relate to suicidal ideation on the MFQ), will undergo a safety assessment with the Columbia Suicide Severity Rating Scale (C-SSRS) [46]. Measure details can be found in Appendix C.

##### Core component 2. Education

All participants and caregivers will be offered a one-time multi-family psychoeducation session, called Mood Foundations. The clinician will provide information on the nature of depression, improving sleep quality, increasing exercise, and healthy diet.

##### Core component 3a. Psychotherapy options: cognitive behaviour therapy

Clinicians will offer participants up to 16 sessions of individual or group-based CBT. The CARIBOU CBT manual is an updated version of the 16-session Lewinsohn and Clarke’s Coping With Depression for Adolescent course [47, 48]. If suicidal ideas and/or self-harming behaviours are present at the assessment, CBT-informed approaches to the management of these symptoms will also be offered using a manualized guide.

##### Core component 3b. Psychotherapy options: brief psychosocial intervention

Adolescents who do not to respond to CBT (that is, less 40% improvement in symptoms on the MFQ over 8 weeks) or report that CBT is not a fit for them, will be offered up to 12 sessions of individual “Brief Psychosocial Intervention” (BPI). BPI involves supportive and pragmatic approaches to address factors thought to be contributing to the adolescent’s depression as per the formulation [49].

##### Core component 4. Caregiver support

Clinicians will offer the youth’s caregivers an 8-session intervention of CBT-based strategies for with respect to communication and problem-solving with adolescents with depression [50].

##### Core component 5. Medication options

For adolescents who initially present with severe depression (MFQ item-mapping onto DSM-5 criteria—see Appendix E of Courtney and colleagues 2019 [51]—clinical impression, and/or presence of self-harm or suicidal ideation), psychiatry appointments will be offered. Psychiatry appointments will also be offered to adolescents who are not responding to 8 weeks of psychotherapy. If medication is warranted, the psychiatrist will follow the medication stream flow diagram recommended by the relevant National Institute of Health Care Excellence guideline [52] and the results of a recent Cochrane meta-analysis [53].

##### Core Component 6. MBC Team Review

Measurement-based care team reviews will consist of (i) completion of self-report measures by the adolescent via an online portal, and (ii) team reviews. The measurement-based care package includes the same measures included in the Core Component 1 initial youth assessment: MFQ, RCADS-anx-25, CADLIS-Y, PGI (Improvement and Severity subscales), and GBO. The primary clinician and other involved health professionals (e.g., psychiatrist, nurse, or social worker), the youth, and ± caregiver(s)) will meet every 4 weeks throughout the intervention to discuss the change-scores in the measurement-based care package and decide to continue or change the current treatment plan at the indicated decision points (i.e., shared decision-making). As with Component 1, adolescents indicating a risk of suicide will undergo a safety assessment including the administration of the C-SSRS by a clinician.

##### Core Component 7. Graduation

A final meeting will be held with the adolescent, relevant clinicians and if the adolescent agrees, caregivers. A summary of the treatment received, a plan for ongoing support and signs of relapse will be discussed. A client-oriented discharge summary (also called a “patient-oriented discharge summary”) will be provided to the youth at the final session; this is an “individualized discharge tool with guidelines that was co-designed with [clients] and families to enable a [client]-centred process” [54].

Acceptable adaptations to how each of these components is provided or executed are available in Table 1. There are no restrictions on other treatments youth participants may receive; any treatments outside of this protocol will be collected and coded using the Health and Social Service Utilization interview (see Appendix C) [55].

**Table 1:** Acceptable adaptations of the CARIBOU-2 pathway components

Component	Individual or Group?	In-person or Online?
1. Assessment	Individual (± Caregiver)	Either
2. Education	Either Individual or Group	Either
3a. Cognitive Behavioural Therapy	Either Individual or Group	Either
3b. Brief Psychosocial Intervention	Individual	Either
4. Caregiver group	Either Individual or Group	Either
5. Medication	Individual (± Caregiver)	Online
6. Team Review	Individual (± Caregiver)	Either
7. Relapse prevention/ Pathway graduation planning	Individual (± Caregiver)	Either

Participants may receive the CARIBOU-2 intervention for up to 52 weeks to allow for all treatment components to occur. A checklist of treatments received will be completed by the research assistant using chart review to document CARIBOU-2 components received. As per the intent-to-treat principle, participants may leave the intervention early and still have the scheduled follow-up research visits.

### Data collection 

Distinct from the measurement-based care package (which are part of the intervention), research measures are also collected to test our study hypotheses, and explore predictors, moderators and mediators of outcome. Research measure results are not provided to study participants (adolescents, caregivers or clincians). Self-report research measures will be captured using REDCap software surveys sent electronically through email to be completed by youth, caregivers and clinicians in the community (e.g. at home or the office) [56]. Evaluator-rated research measures will be completed through semi-structured interviews administered via videoconference (Webex by Cisco) by trained research staff. Research assistants will input ratings directly into REDCap during or immediately following the interview. Diagnostic assessments will be reviewed with the research staff, including the Principal Investigator to ensure reliability among team members. All data will be stored on a password-protected and secure drive at the Centre for Addiction and Mental Health (CAMH). Data quality checks will be conducted yearly by the statisticians on the research team.

Youth will be compensated for their time in research-specific assessments with gift cards valuing $25 CAD to $50 CAD depending on the time point and length of assessment. Research staff will email youth participants at follow-up points, and if they do not respond, participants will attempt a different mode of contact (e.g., phone call or text).

Training of research assistants in interviews will be conducted by a Masters-level research manager/co-ordinator and the Principal Investigator. Inter-rater reliability of the primary outcome measure (CDRS-R) will be assessed on 55 participants in the initial phase of the study, where the lower limit of the 95% confident interval for the intraclass correlation is expected to be ≥ 0.70 to be considered adequate [57].

### Research Measures: Clinical outcomes

Measurement properties, reporting plans and the analysis strategy for all outcome measures are detailed in Appendix C. See Table 2 for the schedule of assessments, including outcome domains, outcome measurement instruments and corresponding informants. The primary outcome measure is the CDRS-R, as rated by a research assistant, blind to treatment arm and study design. Blinded research assistants will be asked to guess to which treatment arm the adolescent was assigned for the purposes of checking the blind. The CDRS-R will be administered at baseline and weeks 4, 12, 24, 36 and 52. Due to the pragmatic nature of the study, participants and co-investigators cannot be blinded to the treatment arm.

**Table 2:** Schedule of assessments for the clinical trial

Domain	Outcome Measurement Instrument	Rater	Follow-up Time Point (Weeks)					
			**0**	**4**	**12**	**24**	**36**	**52**
Demographics	Locally-developed Demographics Form	Youth	ALL					
Diagnosis	K-SADS-PL DSM-5	Evaluator	ALL					
Depression Symptom Severity	CDRS-R	Evaluator	ALL	ALL	ALL	ALL	ALL	ALL
	MFQ	Youth	TAU^a^	TAU^a^	TAU^a^	TAU^a^	TAU^a^	TAU^a^
Depression Diagnosis	DRS	Evaluator	ALL^b^		ALL	ALL	ALL	ALL
Overall mental health	CBCL	Caregiver	ALL			ALL		ALL
Function	CADLIS	Youth	TAU^a^	TAU^a^	TAU^a^	TAU^a^	TAU^a^	TAU^a^
Function	CADLIS	Caregiver	ALL					
Quality of Life	YQOL-R	Youth	ALL		ALL	ALL	ALL	ALL
Self-Injurious Thoughts and Behaviours	Lifetime C-SSRS	Evaluator	ALL					
	Past 6-month C-SSRS	Evaluator	ALL			ALL		ALL
	Lifetime SITBI-NSSI	Evaluator	ALL					
	Past 6 month SITBI-NSSI	Evaluator	ALL			ALL		ALL
Global Impression	CGI-Improvement	Clinician				ALL		
	PGI-Severity	Youth	TAU^a^	TAU^a^	TAU^a^	TAU^a^	TAU^a^	TAU^a^
	PGI-Improvement	Youth		TAU^a^	TAU^a^	TAU^a^	TAU^a^	TAU^a^
Anxiety Symptom Severity	RCADS-15-Anx	Youth	TAU^a^	TAU^a^	TAU^a^	TAU^a^	TAU^a^	TAU^a^
Symptoms of Borderline Personality Disorder	CI-BPD	Evaluator	ALL					
Hopelessness	BHS	Youth	ALL					
Adolescent-Caregiver Conflict	CBQ	Youth	ALL			ALL		ALL
	CBQ	Caregiver	ALL			ALL		ALL
Substance Use	AADIS grid	Youth	ALL					
Health Service Use	Past 3 month HSSU	Evaluator	ALL		ALL	ALL	ALL	ALL
Shared Decision-Making	Collaborate	Youth		ALL	ALL	ALL	ALL	ALL
Service Satisfaction	OPOC-MHA	Youth			ALL	ALL	ALL	ALL
CBT Skill Use	CBTSQ	Youth	ALL	ALL	ALL	ALL	ALL	ALL
COVID restrictions	COVID-Impact	Youth	ALL		ALL	ALL	ALL	ALL

*K-SADS-PL* Kiddie Schedule for Affective Disorders and Schizophrenia- Life-Time Version [58], *CDRS-R* Childhood Depression Rating Scale -Revised [31], *MFQ* Mood and Feelings Questionnaire [32], *DRS* Depression Rating Scale (module within KSADS [58]), *CBCL* Childhood Behavior Checklist [34], *CADLIS-Y* Childhood Anxiety and Depression Life Interference Scale - Youth report [33], *YQOL-R* Youth Quality of Life – Revised [59, 60], *C-SSRS* Columbia Suicide Severity Rating Scale [46], *SITBI-NSSI* Self-Injurious Thoughts and Behaviours Interview – Non-Suicidal Self-Injury subsection [61], *CGI* Clinical Global Impression Scale [44], Patient Global Impression Scale [44], *RCADS-15-Anx* Revised Children’s Anxiety and Depression Scale – 15 item anxiety subscale [43], *CI-BPD* Childhood Interview for Borderline Personality Disorder [62], *BHS* Beck Hopelessness Scale [63], *CBQ* Conflict Behaviour Questionnaire [64], *AADIS* Adolescent Alcohol and Drug Involvement Scale [65], *HSSU* Health System Service Utilization [55], CollaboRATE [66], *OPOC-MHA* Ontario Perception of Care Tool for Mental Health and Addictions [67], *CBTSQ*-Cognitive Behavior Therapy Skills Questionnaire [68], *COVID-Impact* Locally-developed COVID Questionnaire regarding impact of COVID restrictions

^a^To manage respondent burden in CARIBOU-2 pathway arm, these self-report research measures will only be completed by youth in Treatment as Usual arm as the youth in the pathway arm will be completing these measures as part of the measurement-based care package

^b^Embedded in KSADS

To describe the sample of youth participants at baseline, we will capture demographics, diagnosis [58, 62], levels of hopelessness [63], and substance use frequency (which can occur even if severe substance use disorder is an exclusion criterion) [65]. These measures can be used to compare samples across studies and as potential predictors and moderators of response to treatment in secondary analyses [19]. Longitudinal secondary outcomes will assess the extent to which the intervention impacts clinical areas of concern, including depressive symptoms (reported by adolescent [32] and caregiver [69]), depression diagnosis [58], anxiety [43, 69], global impression of overall mental health and improvement [44], self-injurious thoughts and behaviours [46, 61], and caregiver-youth conflict [64]. Measures of shared decision-making [66] and CBT skill use [68] will also be administered to assess potential mechanisms of action of the pathway. Measures of quality of life [59, 60] and health service utilization, with both direct and indirect costs [55], will be used to support the economic evaluation (see below). A measure of service satisfaction will also be captured [67]. Our data collection methods described above will also account for the systematic data collection for significant adverse events, such as, psychiatric hospitalizations, suicide attempts with potential for high lethality or completed suicides.

To explore adolescents’ experiences of the CARIBOU-2 intervention, including acceptability of both the ICP and its components for adolescents, qualitative semi-structured interviews and/or focus groups will be conducted with youth and potential negative effects of psychotherapy. This information will be used to inform future iterations of the ICP and potentially to provide guidance for implementation of the ICP and/or its components. A protocol detailing sub-sample selection, the interview and/or focus group guides and a description of the adolescent perspective component will be published separately.

### Research Measures: Implementation outcomes

Clinician fidelity to the implementation process, the overall ICP and each component of the ICP will be measured using evaluator-rated locally-developed checklists and chart review (see Appendix D for more details on Implementation Outcomes). The exceptions are for CBT and BPI, where the Cognitive Therapy Rating Scale -Revised [70] and Brief Psychosocial Intervention Adherence Scale [8] will be rated by research assistants of randomly selected recordings of therapy sessions. Acceptability of the CARIBOU-2 pathway to the agency staff, as well as barriers and facilitators of implementation will be explored through qualitative interviews with site clinicians guided by the Consolidated Framework for Implementation Research (CFIR) [71].

### Statistical plan

#### Clinical effectiveness analysis

Descriptive data analysis will examine the distribution of collected measures and whether there are significant differences across the treatment arms in participating sites. Missing data patterns and outliers will be carefully examined to provide insight for subsequent analyses. Generalized linear mixed-effects models will be the primary analytic tool for evaluating whether the CARIBOU-2 intervention is more effective than TAU for adolescents with depression presenting to care in the community with regards to improvement of depressive symptoms (Hypotheses A, primary analysis). Secondary outcomes of self-reported depressive symptoms and functioning (Hypotheses B and C), caregiver-reported internalizing psychopathology (Hypothesis D), and suicidal ideation and behaviours (exploratory) will be analyzed with the same method. A generalized linear mixed-effects model controls for covariates (e.g., demographics and baseline clinical measures), accommodates multiple forms of the outcome (e.g., continuous, categorical and count type), and clustering at individual (for repeated measures) and site levels. Time, treatment, and their interactions will serve as the primary predictors for the analyses. We will adopt the intention-to-treat approach and use multiple imputation methods as the primary missing data strategy, with the assumption that data will be missing at random. We anticipate minimal missing data on our primary outcome. In our pilot study, we collected 83% of the expected longitudinal data points on the CDRS-R (primary clinical outcome) [7]. The software package for this project will be R version 4.3.1. No interim analyses on longitudinal research outcomes are planned to limit the possibility of Type I error in testing our primary hypothesis [72]. Using findings of our published scoping review [19], exploratory analyses will be conducted to assess models of prediction, moderation or mediation of outcome. These analyses will be planned and posted on Open Science Framework prior to data collection completion, with the aim to minimize the risk of Type I error through multiple testing [72, 73]. NVivo software will be used to code transcripts of focus groups and qualitative interviews with adolescents. Thematic analysis, described by Braun and Clarke, will be undertaken [74].

#### Power calculation

We anticipate a sample size of 300 adolescent participants. The proposed cluster controlled clinical trial design contains six sites, each with 50 adolescents (25 assigned to the CARIBOU-2 pathway and 25 assigned to TAU), will provide sufficient power (0.80) to detect a small to moderate effect size of 0.40, which is in line with the anticipated effect size from similar existing studies [25, 26]. We calculated power using a Monte Carlo study with 50,000 replications to simulate our unique design. We also conservatively used 0.119 as the site level intra-class correlation and a 20% attrition rate, based on our pilot study [7]. We focused the power calculation on the primary outcome with a pre-post analysis.

We anticipate that about 200 caregivers will participate based on a participation rate of about 66% of adolescents’ caregivers in our pilot study [7]. We anticipate that 70 tertiary participants (site operational implementation team members, supervisors, and clinicians) will participate across 6 sites (some may only be involved in the delivery of TAU).

#### Implementation outcomes analysis

Quantitative implementation outcomes will be analyzed using descriptive statistics, including proportions and distribution of fidelity checklist scores (Hypotheses E, F, G). Qualitative analyses will be used to assess the acceptability of the intervention to agency staff as well as determinant factors that facilitated or hindered implementation based on CFIR 1.0 outlined by Damschroder and colleagues [71]. NVivo software will be used to code transcripts of qualitative interviews. Thematic analysis will be applied to qualitative data.

### Economic evaluation

One of the objectives of the trial is to determine the cost-effectiveness of the CARIBOU-2 pathway compared to TAU. To that end, we will perform a cost-effective analysis and a cost-utility analysis. Results may be site dependent. Details about the economic evaluation of the CARIBOU-2 pathway will be published in a separate protocol.

### Strengths and limitations

Hybrid effectiveness designs enable the simultaneous evaluation of clinical, implementation, and systemic outcomes [37]. Our approach is innovative with respect to the implementation process typical of most randomized trials. Usually, sites are selected early, often prior to funding attainment. Often, selection is solely informed by site willingness at the leadership level and the availability of cases. This approach bypasses important implementation planning in the Exploration phase [39], when organizations explore interventions that might both meet their needs and be feasible to implement in their setting prior to deciding to implement (the concept of adoption) [75]. Many effectiveness trials also miss the early Preparation stage when organizations examine what they must have in place to provide the core components of the target intervention. As described in the methods, our site selection process overcame these limitations.

Our youth engagement approach is also an important innovation within this trial. Youth partners have been involved from the initial intervention design, pilot study and current study [38]. Their involvement optimizes the chances that our results will be relevant to their perspective.

The non-randomized allocation of treatment is a limitation as confounders can readily bias results. While randomized assignment is preferred, our trial design required a pragmatic approach to support logistical aspects of implementation, as well as minimizing the chances of contamination effects. An important limitation of our trial design is that time is a confounder. For example, critical global events (e.g., a pandemic) occurring during the trial could affect outcomes across participants and sites differently depending on the time they entered the trial. The design has limited ability to control for time as a confounder. Another limitation is that, if results are consistent with our hypotheses, we will not be able to discern which pathway components are most important for effectiveness. Follow-up research will be needed to determine the relative importance of each component. Lastly, as with any controlled trial, there is risk of ascertainment bias and non-random attrition from the study (e.g., participants willing to participate throughout the study may differ from those who decline or do not continue).

### Ethics, monitoring and dissemination

#### Ethics, data safety monitoring, auditing and data sharing

Approval has been obtained at the REBs associated with CAMH, The Hospital for Sick Children, and the community-based study sites. All participants will need to provide informed consent for their data to be analyzed and reported (see Appendix E for a copy of the consent form). Data will be de-identified and coded with a unique participant identification number. Three independent scientists external to CAMH have agreed to be on the Data Safety Monitoring Board (DSMB), including one clinical trialist and two psychiatrists. The DSMB charter can be found in Appendix F. Adverse events (psychiatric hospitalizations, suicide attempts with potential for high lethality, completed suicides, death by any cause) will be documented in an Adverse Event Log immediately upon notification and duly reported to the Research Ethics Boards (REBs) and DSMB. Ancillary and post-trial care will be provided by usual services available from public and private means typically available to participants. Any major changes to the protocol will be reported to the REB and DSMB and posted on Open Science Framework. There are no planned audits for the trial.

To promote open science, data-sharing agreements can be made with other research groups within the limits of consent forms for each participant type (youth, caregiver, clinician). Co-investigators will have access to the trial data set with the agreement of the steering committee. Data-sharing agreements will need to be in accordance with up-to-date data governance guidelines, with the aim of supporting the values of participating community agency sites as well as racialized or marginalized communities [76, 77]. Statistical analysis code can be shared upon request to the steering committee.

#### Dissemination

We will create a youth and caregiver friendly knowledge translations product using plain language. The nature of this product (social media, written summary, or other) will be guided by our youth engagement team. Results will be published in a relevant scientific journal with open access and presented at international conferences. Authorship of papers using this data will follow standards set out by the International Committee of Medical Journal Editors [78].

Should our findings show that the CARIBOU-2 intervention is more effective than TAU in reducing depressive symptoms and that it can be implemented with fidelity and adds value to patient care with available resources will inform future efforts to scale up the intervention at other centres. Should our results fail to show differences between TAU and the CARIBOU-2 intervention, whether these relate to failures of the intervention and/or its implementation, then further adaptation to the intervention and/or the implementation approach will be required, along with further effectiveness testing. The economic evaluation will inform policy makers on the value of the pathway with respect to costs. The hybrid effectiveness-implementation design and quasi-experimental cluster design are relatively novel research approaches in child and youth mental health. Our findings will inform future trial designs for complex interventions and implementation research by highlighting the barriers and facilitators of implementing evidence-based interventions in community mental health settings.

### Supplementary Information

** Additional file 1: Appendix A.** Screening Assessment to determine inclusion/exclusion criteria.

** Additional file 2: Appendix B.** Therapy Type Coding for Clinicians delivering Treatment-as-usual (Version 1.0 December 15th 2021).

**Additional file 3: Appendix C.** Properties of Outcome Measurement Instruments and additional Schedules of Assessments.

**Additional file 4: Appendix D.** Implementation outcomes.

**Additional file 5: Appendix E.** Informed consent to participate in a research study: youth version.

**Additional file 6: Appendix F.** The Data Monitoring Committee Charter.

## Data Availability

To promote open science, data-sharing agreements can be made with other research groups within the limits of consent forms for each participant type (youth, caregiver, clinician). Co-investigators will have access to the trial data set with the agreement of the steering committee. Data-sharing agreements will need to be in accordance with up-to-date data governance guidelines, with the aim of supporting the values of participating community agency sites as well as racialized or marginalized communities. Statistical analysis code can be shared upon request to the steering committee. Clinical materials are being rolled out as available for public use at: https://www.camh.ca/en/professionals/treating-conditions-and-disorders/caribou. Shareable research materials are available and routinely updated at: https://osf.io/6qzt7/.
