# Supplementary material for: An Integrated Care Pathway for depression in adolescents: protocol for a Type 1 Hybrid Effectiveness-implementation, Non-randomized, Cluster Controlled Trial
Source: BMC Psychiatry. 2024 Mar 8;24:193. doi: 10.1186/s12888-023-05297-4 (PMC10921633; doi:10.1186/s12888-023-05297-4)
Supplement: Supplementary file 1 — Additional file 1: Appendix A. Screening Assessment to determine inclusion/exclusion criteria. [file 12888_2023_5297_MOESM1_ESM.docx]

Appendix A. Screening Assessment to determine inclusion/exclusion criteria

| Screening Question: | If the following is true, the **youth is not eligible and the subsequent screener questions will not appear.** |
| --- | --- |
| Agency: |  |
| Has the youth been assigned a Clinician? |  |
| Please provide us with the Clinician's Initials: |  |
| Date: |  |
| Youth's Initials: |  |
| Youth's Chart #: |  |
| Youth's current gender identity: | N/A |
| Youth's age: | Youth’s age is less than 13 or greater than 18 |
| Youth and/or caregiver is expressing that "depression" is a concern? | No |
| Clinician agrees that depressive symptoms are a treatment target? | No |
| Youth is fluent in English (i.e., comprehension, read, write, communication)? | No |
| Youth is new to receiving treatment at the agency within the past 3 months? | N/A |
| Youth has had a period of 3 months or more of treatment at the agency within the past 6 months ? | Yes |
| Youth is attending, or will soon be attending, a Day Treatment Program? | Yes |
| Youth has a known or highly suspected... Intellectual disability? | Yes |
| Youth has a known or highly suspected... Presentation of psychotic symptoms that are persistent and have observable effects on behaviour (i.e., consistent with schizophrenia)? | Yes |
| Youth has a known or highly suspected... Severe substance use disorder (e.g., daily cannabis use throughout the day, heavy alcohol/ Benzodiazepines use 3 or more times a week, cocaine/opiate use several days a month or more)? | Yes |
| Youth has a known or highly suspected... Bipolar disorder (e.g., elevated mood and energy outside typical presentation and changes observable by others lasting for 4 consecutive days or more)? | Yes |
| Youth has a known or highly suspected... Severe eating disorder (e.g., restrictive eating patterns, associated with preoccupation with body image, leading to weight loss and associated medical complications and/or bingeing and purging at least 2 times a week)? | Yes |
| Youth is at imminent risk of suicide requiring hospitalization as per judgment of the assessing clinician? | Yes |
| Youth is able to provide informed consent to the study for any reason (e.g., there is no language barrier, nor intellectual disability, nor severe psychosis that would be a barrier to informed consent)? | No |
| Youth's MFQ score: | MFQ score is less than 22 |
| Youth is agreeable to be contacted by RA to describe the project? | No |
