# Supplementary material for: An Integrated Care Pathway for depression in adolescents: protocol for a Type 1 Hybrid Effectiveness-implementation, Non-randomized, Cluster Controlled Trial
Source: BMC Psychiatry. 2024 Mar 8;24:193. doi: 10.1186/s12888-023-05297-4 (PMC10921633; doi:10.1186/s12888-023-05297-4)
Supplement: Supplementary file 2 — Additional file 2: Appendix B. Therapy Type Coding for Clinicians delivering Treatment-as-usual (Version 1.0 December 15th 2021). [file 12888_2023_5297_MOESM2_ESM.docx]

Appendix B. Therapy Type Coding for Clinicians delivering Treatment-as-usual (Version 1.0 December 15^th^ 2021)

| Cognitive Behavioural Therapy | - Setting an agenda - Establishing connections between recent thoughts, behaviours and emotions - Establishing connections between antecedents, behaviours and consequences of behaviour patterns - Psychoeducation regarding learning theory - Behavioural activation and activity scheduling - Assigning behavioural experiments - Exposure/systematic desensitization - Assigning cognitive restructuring tasks - Physical interventions (progressive muscle relaxation; abdominal breathing) |
| --- | --- |
| Psychodynamic Psychotherapy | - Inferring unconscious motives for behaviours - Identifying defense mechanisms - Discussing wishes, fantasies, dreams or early childhood memories - Examining transference and countertransference |
| Narrative therapy | - Psychoeducation that life as containing multiple truths and that people are the experts in their own lives - Looks at dominant narratives and “thickens” preferred or alternative narratives - Deconstructing complex life events - Guiding client to more adaptive (and non-judging) understanding of life story |
| Emotion-focused (family) therapy: | - Reframing unpleasant emotions as important information and potentially helpful - Increasing client’s understanding of their own emotions (identification, labelling) - Validating the emotion - Increasing clients ability to accept emotional experience - Increasing client’s awareness of multiple layers of emotions - Increasing client’s ability to evaluate emotions as helpful or unhelpful and direct action accordingly - Develop personal scripts to challenge destructive thoughts - Coaching on how to meet the need of the emotion - Teaching parenting strategies for connecting with children such as emotion and behaviour coaching, validation and therapeutic apology - Use of role plays to practice scripts for exploring emotions |
| Dialectical Behaviour Therapy | - Psychoeducation on the biosocial theory - Teaching skills related to mindfulness, distress tolerance, emotion regulation and interpersonal effectiveness. - Using behavior chain analyses - Addressing treatment-interfering behaviour - Dialectical strategies |
| Third-wave strategies | - Mindfulness exercises - Acceptance strategies - Exploring values - Value-driven goals and behaviours |
| Solution-focused therapy | - Exploring progress client has already made and how to build on this - Exploring the clients’ ideal life circumstance - Discussing ways to obtain this life circumstance - Use of scaling questions, miracle question and other tools for assisting in achieving goals |
| Motivational Interviewing | - Asking open-ended questions, providing affirmations, engaging in reflective listening and providing summaries of understanding - Expressing empathy, Rolling with Resistance, Developing Discrepancy, Supporting Self-efficacy |
| Creative Arts Therapy | - Use of an artistic medium (art, music, dance, poetry) for self-expression, developing insights, or problem-solving |
| Attachment-based treatment | - Exploring early childhood attachment issues - Identifying which processes were suppressed/overemphasized - Creation of a secure base to explore and respond safely - Increasing opportunity for choice and empowerment - Psychoeducation around ARC therapy goals - Establishing a routine |

| Structure of Session | - Client only - Client and caregivers - Caregivers only - Client and caregivers sequentially - Other family members (siblings) - Group |
| --- | --- |
