# Supplementary material for: An Integrated Care Pathway for depression in adolescents: protocol for a Type 1 Hybrid Effectiveness-implementation, Non-randomized, Cluster Controlled Trial
Source: BMC Psychiatry. 2024 Mar 8;24:193. doi: 10.1186/s12888-023-05297-4 (PMC10921633; doi:10.1186/s12888-023-05297-4)
Supplement: Supplementary file 3 — Additional file 3: Appendix C. Properties of Outcome Measurement Instruments and additional Schedules of Assessments. [file 12888_2023_5297_MOESM3_ESM.docx]

**Appendix C: Properties of Outcome Measurement Instruments and additional Schedules of Assessments.**

**CARIBOU-2 Intervention Measurement-Based Care Measures:**

These measures are fed back to clinicians and youth participants in the CARIBOU-2 intervention arm just prior to team reviews every 4-weeks as an integral part of the CARIBOU-2 intervention as part of measurement-based care (MBC). The CARIBOU-2 pathway suggests treatment options shared decision-making around treatment changes occur based on the measures, youth participant values as well as clinician expertise.

1. **The Mood and Feelings Questionnaire child, long version (MFQ; Angold & Costello, 1987)**^1^**:** The MFQ is a 33-item self-report measure for youth regarding depressive symptoms experienced over the prior 2 weeks. It was specifically recommended in the NICE guideline, given its psychometric properties; namely, it performed well as a discriminator of an affective disorder (AUC 0.93, 0.90-0.96 for a cut-off score of ≥22)^2^, has good internal consistency (α=0.92-0.94)^2^ and good test-retest reliability (Pearson’s r=0.78)^3^. The MFQ score will be dichotomized to indicate clinical levels of depression with a score of ≥22. It will also be used as a continuous measure to indicate improvement or deterioration within the MBC framework.
2. **Revised Children's Anxiety and Depression Scale (RCADS; Chorpita et al, 2000)**^4^**:** In our CARIBOU-1 pilot study, 69% of youth entering the intervention also met criteria for an anxiety disorder.^5^ Treatment of anxiety overlaps substantially with treatment of depression (including the use of Selective Serotonin Reuptake Inhibitors and Cognitive Behavioural Therapy), so targeting these symptoms, along with depression, is logical to pursue. We will use the 15-item anxiety subscale of the 25-item version of the RCADS^4^. After a thorough review, the RCADs were recommended by the International Consortium for Health Outcomes Measurement (ICHOM) for clinical use due to its feasibility and reported measurement properties^6^. RCADS t-scores can be used by clinicians to classify symptoms on an ordinal scale of “non-clinical” (t-score <65), “threshold” (t-score 65-69) and “clinical” (t-score ≥70). Raw scores and t-scores can also be used as a continuous measure to capture change over time.
3. **The Child Anxiety and Depression Life Interference Scale-Youth Version (CADLIS):** The CADLIS is a modified version of the anxiety-specific measure, Child Anxiety Life Interference scale^7^, which was recommended by ICHOM^6^. The modified version contains 10-items that ask about impact of both depression and anxiety on facets of functioning. All items, which relate to common activities (e.g. “being with friends outside of school”), are rated on a five-point Likert scale (0 = not at all, 4 = a great deal), with higher scores indicating higher anxiety life interference. This measure was chosen by our youth partners due to its content validity and acceptability. The CADLIS can be used by clinicians as a continuous measure to capture change over time. Clinicians may also wish to follow-up with youth participants around specific areas of life interference looking at each item individually.
4. **Patient Global Impression-Improvement Subscale (PGI-I; Guy, 1976)**^8^**:** The Patient Global Impression of Improvement Scale was developed by the NIMH in 1976. It is composed of 1-item which asks how the patient is feeling since a given time point (i.e., since starting at the clinic, since last major treatment change, or since last minor treatment change). There are seven options for the patient to select and are scored from 1 to 7, respectively: "very much better", "much better", "a little better", "no change", " a little worse", "much worse", "very much worse". The stem and response correspond to the widely used Clinical Global Impressions - Improvement Scale^8^. It is not applied at baseline, as the question is with reference to how the youth was doing at baseline. Clinicians can use the PGI-I as a single item continuous measure.
5. **Goals Based Outcome (GBO, Law and Jacob, 2015)**^9^**.** The Goals Based Outcome is a measure used to identity and track progress on personalized goals. As it is an idiographic measure, measurement properties need to be considered differently that typical self-report clinical measures. Duncan and colleagues have examined measurement properties and report adequate stability of goals over a period of 6 to 24 weeks, moderate convergent validity with other measures of mental health and sensitivity to change to a psychological intervention^10^.
6. **The Columbia Suicide Severity Rating Scale (C-SSRS, Posner et al)**^11^**:** This measure contains items related self-injurious thoughts and behaviours. Interviewers rate 3 subscales: Suicidal Ideation Severity, Suicidal Behaviour (which includes suicide attempts and an item for non-suicidal self-injury that is recorded as distinct from suicidal behaviour), and Lethality. Favourable psychometric properties have been described in both adolescent and young adult samples^12^. When used as a part of measurement-based care, the frame of reference is “since last assessment”. Active planning of suicide (score of 4 or 5 on the suicidal ideation subscale) or recent suicide attempt would prompt further acute safety management which may involve consultation with senior staff at the agency as well as engagement of caregivers in safety planning. The Suicidal Ideation Severity and Lethality subscales can be used as a continuous measure. The Suicidal Behaviour subscale is used as dichotomous measures for each item (i.e., present or absent within the time interval of interest).

Table S1 outlines details regarding the timing of the measurement-based care measures.

**Table S1. Schedule for Measurement-Based Care Measures completed for Youth Participants receiving the CARIBOU-2 intervention.**

|  |  |  | **Week** | | | | | | | | | | | | | |
| --- | --- | --- | --- | --- | --- | --- | --- | --- | --- | --- | --- | --- | --- | --- | --- | --- |
| **Measures** | **Time (mins)** | **Informant** | **0** | **4** | **8** | **12** | **16** | **20** | **24** | **28** | **32** | **36** | **40** | **44** | **48** | **52** |
| MFQ* | 5 | Youth | x | x | x | x | x | x | x | x | x | x | x | x | x | x |
| RCADS-25 | 3 | Youth | x | x | x | x | x | x | x | x | x | x | x | x | x | x |
| CADLIS | 3 | Youth | x | x | x | x | x | x | x | x | x | x | x | x | x | x |
| PGI-I | 1 | Youth |  | x | x | x | x | x | x | x | x | x | x | x | x | x |
| GBO | 2 | Youth | x | x | x | x | x | x | x | x | x | x | x | x | x | x |
| C-SSRS^a^ | 5 | Clinician | x | x | x | x | x | x | x | x | x | x | x | x | x | x |

*includes 4 items related to suicidal ideation

^a^Only completed if MFQ indicates some suicidal ideation.

NB: If there is 8 weeks of remission (MFQ<22), participant may discontinue MBC measures while continuing research measures and still be considered adherent to the research protocol.

At the beginning of the assessment package, youth will be made aware that the results of these assessments are not monitored in real-time and that if they consider themselves to be a risk to their own safety or the safety of someone else, to consider going to the emergency room for assessment.

**CARIBOU-2 Youth Participant Research Measures of Clinical Outcomes**

These measures will be completed by youth in both arms of the study (i.e., CARIBOU-2 intervention and TAU). They will be used to test our hypotheses and exploratory aspects with respect to clinical effectiveness of the CARIBOU-2 intervention and potential moderators of outcome, assuming it is delivered with good fidelity. Blinding the treatment the participants and clinicians receive is not feasible. The RA conducting the primary outcome measure (CDRS-R) will be blind to the treatment arm.

**Baseline Measures:**

1. **The Mood and Feelings Questionnaire child, long version (MFQ; Angold & Costello, 1987):** As previously described. In addition to an MBC measure, it will also be used as a screening measure to determine eligibility for the study.
2. **Demographic Form:** A standard demographic form will be used to capture multiple aspects of the youth's profile at baseline. These include questions around sex assignment at birth, current gender identity, sexual orientation, caregiving arrangements (i.e., parents/guardians), socio-economic status, and ethnicity.
3. **Kiddie Schedule for Affective Disorders and Schizophrenia- Life-Time Version with respect to DSM-5 (K-SADS-PL DSM-5; Kaufman et al., 2000)**^13^**:** The KSADS is a semi-structured interview that combines dimensional and categorical assessment approaches to diagnose current and past episodes of psychopathology in children and adolescents (ages 6-18 years old) modified to refer to DSM-5 criteria. This measure will be used to describe the participants in the study.
4. **The Childhood Interview for Borderline Personality Disorder (CI-BPD)**^14^**:** This is a semi-structured RA-rated interview which assesses for the 9 DSM-IV-TR criteria for Borderline Personality Disorder (identical to DSM-5 criteria) requiring that symptoms be “definitely present” for 2 or more years to be considered to meet the threshold. It has been validated in adolescent samples^14^. It will be collected to describe our baseline sample and explored as a potential predictor or moderator of treatment outcome. The CI-BPD results will be reported as dichotomous, indicating either meeting threshold criteria for BPD or not.
5. **Beck Hopelessness Scale-Revised (BHS-R; Neufeld, O'Rourke, & Donnelly, 2010)**^15^**:** A 20-item self-report revision of the original Beck Hopelessness Scale (BHS; Beck & Steer, 1998), that measures negative attitudes about one’s future and perceived inability to avert negative life occurrences. The three aspects of hopelessness (negative feelings about the future; loss of motivation; and pessimistic expectations) are measured on a four-point Likert-type scale. It will be collected to describe our baseline sample and potentially explored as a predictor or moderator of treatment outcome. BHS results will be reported as a continuous measure.
6. **Adolescent Alcohol and Drug Involvement Scale – Substance Use Frequency Grid** (AADIS-Grid, Moberg 2003).This measures asks youth to indicate the frequency of substance use (?over the past year) with response options including “not at all”, “several times a year”, “several times a month”, “weekends only”, “several times a week”, “daily” and “ multiple times a day”. Substances of interest include tobacco, cannabis, alcohol, amphetamines, cocaine, benzodiazepines, opioids, hallucinogens, and other substances. It is used to describe our baseline sample. Results will be analyzed and reported as an ordinal measure.

**Primary outcome measure:**

1. The **Childhood Depression Rating Scale-Revised (CDRS-R)**^16^ is a 17-item measure rated by an RA following a semi-structured interview with the adolescent relating to symptoms of depression over the past 2 weeks. The RA will be blind to treatment assignment and study design. This measure was chosen as youth with lived experience have identified relief of depressive symptoms as a priority outcome domain^17^. The CDRS-R is also the most commonly used measure in RCTs for depression in adolescents, which could assist in comparing results across studies^18^. While there are important limitations regarding the current evidence of measurement properties of the CDRS-R^19^, we are not aware of any evidence of a more robust measure. Mayes and colleagues (2010) found good internal consistency (alpha=0.74-0.92) and correlated significantly with measures of global severity of illness (r=0.80-0.93, p<0.01), functioning (r=0.52-0.77, p<0.01) and a diagnosis of major depressive disorder on the K-SADS-PL (r=0.64, p<0.01). Change in the score was also correlated with measures of global improvement with treatment (r=0.83, p<0.01). In our non-randomized pilot study, 10 CDRS-R interviews were recorded to test for inter-rater reliability; the weighted Cohen’s kappa between the principal investigator and research assistant ranged from 0.76 to 0.92^5^. It is our primary outcome measure to test our primary hypothesis and will be analyzed and reported as a continuous measure.

**Secondary clinical outcome measures**

1. The **Columbia Suicide Severity Rating Scale (C-SSRS)** was described above. In addition to being an MBC measure, it is also an important exploratory outcome with respect to our research. As a research measure, it will be captured at baseline with the highest lifetime rating on each subscale will be obtained as outcomes to explore potential as a risk factor for various outcomes. At baseline and 6-month follow-up, highest rating in the past 6 months on each subscale will also be captured to assess longitudinal patterns.
2. The **Depression Rating Scale (DRS)**^13^ is RA-rated subscale found in the K-SADS-PL DSM-5 used to assess for DSM-5 criteria of Major Depressive Disorder (MDD). The RA will be blind to treatment arm when conducting this assessment. This will be used to compare with the CDRS-R and MFQ over time to examine how much these scale scores overlap with DSM-5 criteria. It will be analyzed and reported as an ordinal outcome: not meeting criteria, mild MDD, moderate MDD, and severe MDD, with reference to DSM-5 definitions for severity categories.
3. **Conflict Behaviour Questionnaire (CBQ)** A 20-item true/false scale that assesses general conflict between parents and their children. The CBQ has been used extensively as a potential predictor of outcome in multiple RCTs of adolescent depression (Brent et al., 1997; Brent et al., 2008; Mufson et al., 2004; March et al., 2004) and has adequate internal consistency (Robin & Foster, 1989). The cut-off reference is a total score of ≥9 (Rengasamy et al., 2013). This measure is only applied at baseline and every 6 months to explore if the caregiver-adolescent conflict changes with treatment and time.
4. The **Youth Quality of Life Scale Research Version (Y-QOL)**^20,21^ is a 41 item self-report scale measuring the broad array of constructs including sense of self-worth, quality of relationships, sense of agency and life satisfaction. The YQOL-R showed reliability, content validity, construct validity and criterion validity^20,21^. This measure will be used as a continuous measure as part of the economic evaluation.
5. The **Health and Social Service Utilization interview HSSU**^22^ is an evaluator-rated semi-structured assessment which quantifies health service utilization data. This includes number of sessions obtained both within and outside the study site, as well as hospital admissions and medication use. This data will be used primarily for the economic evaluation.
6. The **CollaborATE**^15^ is a 3-item self-report measure of shared decision-making developed with patient input, with each item rated on a scale of 0-9. This measure intended to capture variability in shared decision-making between treatment arms and explore shared decision-making as a potential mediator of the CARIBOU pathway. The measure will be reported and analyzed as a continuous measure for the sum score^23^; however, if there are ceiling effects, we will use this as a dichotomous score, where participants are divided into those who respond with the maximum score versus those who do not^23,24^.
7. The **Ontario Perception of Care Tool for Mental Health and Addictions (OPOC-MHA)**^25^ is a self-report measure representing extent of service satisfaction. This asks respondents to rate satisfaction on themes of access to services provided, participation and rights, staff (including therapists), environment, discharge and recovery outcome and service quality. The measure was assessed as having a 2-factor structure based on both exploratory factor analysis and confirmatory factor analysis. The two factors correspond to experience accessing services, and to experience within the service, each with excellent internal consistency (alpha> 0.90)^25^. The data will be reported and analyzed as a continuous sum score for each of the two corresponding subscales as well as the total sum score.
8. **The Cognitive Behaviour Therapy Skills Questionnaire (CBTSQ)**^26^ is a 16-item self-report measure intended to track changes in CBT skill use throughout treatment. This measure was selected, as measures of “coping” were assessed as important by youth samples^17^. This specific measure was chosen over others by our youth engagement team^27^. Jacob and colleagues identified a two-factor structure, with the first subscale measuring behavioural activation strategies, and the second measuring cognitive restructuring strategies; each factor demonstrated good internal consistency (alphas > 0.80)^26^. Increases in skill use have been associated with greater improvement in mood in adults with depression receiving CBT^26^. Results will be used to assess potential mechanisms of change. Each subscale of the CBTSQ will be analyzed and reported as a continuous sum score measure.
9. The **MFQ, RCADS-25-anx, PGI-S, and PGI-I measures** (described above) will be collected at key time points in the TAU group without feedback to clinicians or youth participants. These will allow for comparisons to MBC measures captured in the CARIBOU-2 intervention group.

**Timing of Youth Participant Measures**

Refer to [Table](https://www.ncbi.nlm.nih.gov/pmc/articles/PMC6863574/table/t1-ccap28_p0115/) 2 in the main manuscript for details on the administration of the effectiveness research measures. Customized demographic information and treatment history forms will be administered to youth to describe the basic characteristics of the recruited sample.

The following are justifications for our time points:

- 4-week time point: Early response predicts outcome (Gunlicks Stoessel, 2019).
- 12-week time point: Major studies like TADS (March et al., 2004), TORDIA (Brent et al., 2008), IMPACT (Goodyear, 2017) have this time point to facilitate data comparison.
- 24 weeks is the primary end time point: To allow enough time for youth to proceed through much of the pathway, without concern about contamination effects.
- 36-week time point and 52-week time point: To allow further time to explore effects of the CARIBOU-2 intervention, though contamination effects may be problematic.

**Caregiver Participant Measures**

These measures are administered to participating caregivers (e.g., Parents/Guardians) in both study arms (i.e., TAU and ICP). The data will be used to further test our hypotheses and the exploratory aspects of the clinical effectiveness of the CARIBOU-2 intervention, assuming it is delivered with good fidelity.

**Baseline measures**

1. **Demographic Form:** A standard demographic form will be used to capture multiple aspects of the caregiver's profile at baseline. These include questions around their own sex assignment at birth, current gender identity, sexual orientation, socio-economic status, and ethnicity.

**Secondary clinical outcome measure**

1. **The Child Behaviour Checklist – Parent Report Form (CBCL; Achenbach, 2001):** A 118-item caregiver-rated measure assessing the youth’s behaviour and general psychopathology. It is a widely used measure with known population norms. One-week test-retest reliability was found to be 0.80-0.94. Internal consistency is reported to be high; inter-rater reliability (e.g., between two parents) was found to be moderate to high (reference needed). Continuous t-scores for the total problems scale and all subscales will be used at baseline to describe general psychopathology. The internalizing broadband scale of the CBCL (ie. anxious-depressed, depressed-withdrawn and somatic subscales combined) will be measured longitudinally to get an impression of how the caregiver is observing any changes in mood or anxiety with treatment.
2. **Conflict Behaviour Questionnaire-20-Parent (CBQ-20-P; Robin & Foster, 1989):** A 20-item measure of perceived parent-child communication conflict behavior at home. Parents retrospectively rate (i.e., “True” or “False”) their interactions with their adolescent children over the 2 or 3 weeks preceding the assessment session. The cut-off reference is a total score of 11 or greater (Rengasamy et al., 2013). This measure will be compared with the CBQ as reported by youth participants at baseline to see if there are discrepancies. Follow-up measures will be administered to see if change has occurred over time, particularly for caregivers who participated in the caregiver group component of the pathway. This measure will be reported and analyzed as a continuous sum score.

**Timing of Caregiver Participant Measures** Refer to Table S2 for details on the administration of these caregiver participant research measures.

**Table S2. Schedule of Assessments for Caregiver Participant Research Measures**

|  |  |  | **Weeks** | | | | |
| --- | --- | --- | --- | --- | --- | --- | --- |
| **Measures** | **Rater** | **Time (mins)** | **0** | **4** | **12** | **24** | **52** |
| **Demographic Form** | Caregiver | 5 | x |  |  |  |  |
| **CBCL** | Caregiver | 20 | x |  |  | x | x |
| **CBQ-20-P** | Caregiver | 5 | x |  |  | x | x |

1. Angold A, Costello EJ. Mood and feelings questionnaire (MFQ). *Durh Dev Epidemiol Program Duke Univ*. Published online 1987.

2. Neufeld SAS, Dunn VJ, Jones PB, Croudace TJ, Goodyer IM. Reduction in adolescent depression after contact with mental health services: a longitudinal cohort study in the UK. *Lancet Psychiatry*. 2017;4(2):120-127. doi:10.1016/S2215-0366(17)30002-0

3. Wood A, Kroll L, Moore A, Harrington R. Properties of the mood and feelings questionnaire in adolescent psychiatric outpatients: a research note. *J Child Psychol Psychiatry*. 1995;36(2):327-334.

4. Chorpita BF, Yim L, Moffitt C, Umemoto LA, Francis SE. Assessment of symptoms of DSM-IV anxiety and depression in children: A revised child anxiety and depression scale. *Behav Res Ther*. 2000;38(8):835-855. doi:10.1016/S0005-7967(99)00130-8

5. Courtney DB, Cheung A, Henderson J, et al. CARIBOU‐1: A pilot controlled trial of an Integrated Care Pathway for the treatment of depression in adolescents. *JCPP Adv*. 2022;2(2). doi:10.1002/jcv2.12083

6. Krause KR, Chung S, Adewuya AO, et al. International consensus on a standard set of outcome measures for child and youth anxiety, depression, obsessive-compulsive disorder, and post-traumatic stress disorder. *Lancet Psychiatry*. 2021;8(1):76-86. doi:10.1016/S2215-0366(20)30356-4

7. Lyneham HJ, Sburlati ES, Abbott MJ, et al. Psychometric properties of the Child Anxiety Life Interference Scale (CALIS). *J Anxiety Disord*. 2013;27(7):711-719. doi:10.1016/j.janxdis.2013.09.008

8. Guy W. Clinical global impression scale. *ECDEU Assess Man Psychopharmacol-Revis Vol DHEW Publ No ADM 76*. 1976;338:218-222.

9. Law D, Jacob J. *Goals and Goal Based Outcomes (GBOs) Some Useful Information*. CAMHS Press; 2015.

10. Duncan C, Cooper M, Saxon D. Test–retest stability, convergent validity, and sensitivity to change for the Goal‐Based Outcome tool for adolescents: Analysis of data from a randomized controlled trial. *J Clin Psychol*. 2023;79(3):683-696. doi:10.1002/jclp.23422

11. Posner K, Brent D, Lucas C, et al. Columbia-suicide severity rating scale (C-SSRS). *N Y NY Columbia Univ Med Cent*. Published online 2008.

12. Posner K, Ph D, Brown GK, et al. The Columbia–Suicide Severity Rating Scale: initial validity and internal consistency findings from three multisite studies with adolescents and adults. *Am J Psychiatry*. 2011;168(12):1266-1277. doi:10.1176/appi.ajp.2011.10111704

13. Kaufman J, Birmaher B, Brent DA, Ryan ND, Rao U. K-sads-pl. *J Am Acad Child Adolesc Psychiatry*. 2000;39(10):1208.

14. Sharp C, Ha C, Michonski J, Venta A. Borderline personality disorder in adolescents: evidence in support of the Childhood Interview for DSM-IV Borderline Personality Disorder in a sample of adolescent. *Comprehensive*. Published online 2012. Accessed June 28, 2016. http://www.sciencedirect.com/science/article/pii/S0010440X11002367

15. Elwyn G, Barr PJ, Grande SW, Thompson R, Walsh T, Ozanne EM. Developing CollaboRATE: A fast and frugal patient-reported measure of shared decision making in clinical encounters. *Patient Educ Couns*. 2013;93(1):102-107. doi:10.1016/j.pec.2013.05.009

16. Poznanski EO, Freeman LN, Mokros HB. Children’s depression rating scale, revised (CDRS-R). *Psychol Bull*. 1985;21:979-989.

17. Krause KR, Edbrooke-Childs J, Bear HA, Calderón A, Wolpert M. What treatment outcomes matter most? A Q-study of outcome priority profiles among youth with lived experience of depression. *Eur Child Adolesc Psychiatry*. Published online July 17, 2021. doi:10.1007/s00787-021-01839-x

18. Krause KR, Bear HA, Edbrooke-Childs J, Wolpert M. Review: What Outcomes Count? A Review of Outcomes Measured for Adolescent Depression Between 2007 and 2017. *J Am Acad Child Adolesc Psychiatry*. 2019;58(1):61-71. doi:10.1016/j.jaac.2018.07.893

19. Stallwood E, Monsour A, Rodrigues C, et al. Systematic Review: the Measurement Properties of the Children’s Depression Rating Scale-Revised in Adolescents With Major Depressive Disorder. *J Am Acad Child Adolesc Psychiatry*. Published online 2020.

20. Edwards TC, Huebner CE, Connell FA, Patrick DL. Adolescent quality of life, part I: conceptual and measurement model. *J Adolesc*. 2002;25(3):275-286.

21. Patrick DL, Edwards TC, Topolski TD. Adolescent quality of life, part II: initial validation of a new instrument. *J Adolesc*. 2002;25(3):287-300.

22. Browne GB, Arpin K, Corey P, Fitch M, Gafni A. Individual correlates of health service utilization and the cost of poor adjustment to chronic illness. *Med Care*. Published online 1990:43-58.

23. Ubbink DT, Van Asbeck EV, Aarts JWM, et al. Comparison of the CollaboRATE and SDM-Q-9 questionnaires to appreciate the patient-reported level of shared decision-making. *Patient Educ Couns*. 2022;105(7):2475-2479. doi:10.1016/j.pec.2022.03.007

24. Forcino RC, Barr PJ, O’Malley AJ, et al. Using CollaboRATE, a brief patient-reported measure of shared decision making: Results from three clinical settings in the United States. *Health Expect*. 2018;21(1):82-89. doi:10.1111/hex.12588

25. Rush B, Hansson E, Cvetanova Y, Rotondi NK, Furlong A, Behrooz R. *Development of a Client Perception of Care Tool for Mental Health and Addictions: Qualitative, Quantitative, and Psychometric Analysis: Final Report for the Ministry of Health and Long-Term Care*. Centre for Addiction and Mental Health; 2013.

26. Jacob KL, Christopher MS, Neuhaus EC. Development and Validation of the Cognitive-Behavioral Therapy Skills Questionnaire. *Behav Modif*. 2011;35(6):595-618. doi:10.1177/0145445511419254

27. Prebeg M, Relihan J, Darnay K, et al. Youth Partner Engagement in the Development of an Integrated Care Pathway for the Treatment of Adolescents with Depression. *PsyArXiv Prepr*. doi:https://psyarxiv.com/kyzcj/
