# Supplementary material for: An Integrated Care Pathway for depression in adolescents: protocol for a Type 1 Hybrid Effectiveness-implementation, Non-randomized, Cluster Controlled Trial
Source: BMC Psychiatry. 2024 Mar 8;24:193. doi: 10.1186/s12888-023-05297-4 (PMC10921633; doi:10.1186/s12888-023-05297-4)
Supplement: Supplementary file 4 — Additional file 4: Appendix D. Implementation outcomes. [file 12888_2023_5297_MOESM4_ESM.docx]

**Appendix D Implementation Outcomes:**

To assess the implementation outcomes, assessments at the level of the clinic will be held continually throughout the study period. The implementation outcomes are based on the Implementation Outcome Taxonomy outlined by Proctor and colleagues^1^. Implementation outcomes that will be examined in the current study are feasibility, fidelity, cost-effectiveness, and acceptability. Implementation outcomes that are outside the scope of evaluation for this particular study include: adoption, penetration and sustainability, though some may be explored in future studies.

1. **Fidelity: “**Fidelity is defined as the degree to which an intervention was implemented as it was prescribed in the original protocol or as it was intended by the program developers”.^1^
   1. **Fidelity to the Implementation Process:** For our results to be replicable, the implementation process set out in section 4.2 will need to be followed with fidelity. We have created a checklist for this process to ensure replicability. This fidelity measure is with reference to tasks completed by the implementation teams.
   2. **Fidelity to the Overall CARIBOU-2 intervention:** Fidelity to the overall CARIBOU-2 intervention will be assessed by a checklist for each youth participant as they proceed through the pathway. This fidelity measure is with reference to tasks to be completed by direct clinicians. Items include whether youth were offered the treatment components of the ICP as indicated, whether measurement-based care was offered and whether team reviews were offered every 4 weeks until exit from the pathway. These items are monitoring factors under the clinicians’ control. We anticipate that clinicians will have an average of ≥75% fidelity to the CARIBOU-2 intervention.
   3. **Fidelity to Components of the ICP:** For the following psychosocial intervention fidelity measures, all sessions will be audio recorded. For Mood Foundations, at least 5 sessions will be chosen randomly to be assessed for fidelity. For other psychotherapy sessions, 20% of all sessions will be selected randomly to assess for fidelity. Research assistants will score the fidelity based on the recordings. ≥75% fidelity on average is targeted for each component.

**Fidelity to the Mood Foundations Session, Medication algorithm, and Caregiver support group** will be measured by locally-created checklists, developed a priori.

**Fidelity to CBT** will be assessed using the Cognitive Therapy Rating Scale – Revised^2^. Some items of this scale are not relevant for group therapy, and will be adjusted accordingly for group sessions.

**Fidelity to BPI** will be assessed using the Brief Psychosocial Intervention Adherence Scale^3^. This is an 8-item scale created by the BPI developers. It was also used in the IMPACT trial to assess fidelity to the therapy^4^.

1. **Feasibility:** Feasibility is “the extent to which a new treatment, or an innovation, can be successfully used or carried out within a given agency or setting” (Procter et al, 2011). If youth are attending with the various components of the pathway, assuming fidelity to the model, it can clearly be said that the intervention is feasible. Using a locally-developed youth participant engagement checklist, prospective chart review, and RA communication with clinicians, the proportion of indicated components the youth participant attends will be tracked over time and reported.
2. **Acceptability, barriers and facilitators of implementation:** In order to qualitatively assess acceptability of the CARIBOU-2 intervention as well as facilitators and barriers of implementation, we will assess the 39 implementation constructs from the Consolidated Framework for Implementation Research (CFIR) outlined by Damschroder et al 2009. One-to-one interviews between research staff and clinical staff using the CFIR interview (see Appendix F) will be conducted 1-year after initial implementation to assess these constructs. Three staff at each site, each with high involvement in the delivery of the CARIBOU-2 intervention, will undergo this interview process.

The CFIR is composed of five major domains, each of which may affect an intervention’s implementation:

1. **Intervention characteristics:** Features of an intervention that might influence implementation. It consists of eight constructs (e.g., stakeholders’ perceptions about the relative advantage of implementing the intervention, complexity).
2. **Inner setting:** Features of the implementing organization that might influence implementation. It consists of twelve constructs (e.g., implementation climate, leadership engagement).
3. **Outer setting:** Features of the external context or environment that might influence implementation. It consists of four constructs (e.g., external policy and incentives).
4. **Characteristics of individuals:** The characteristics of the individuals involved in implementation that might influence implementation. It consists of five constructs (e.g., knowledge and beliefs about the intervention).
5. **Implementation process:** Strategies or tactics that might influence implementation. It consists of eight constructs (e.g., engaging appropriate individuals in the implementation and use of the intervention, reflecting, and evaluating).

**Timing of Implementation Outcome Capture:**

Implementation outcomes will follow the timeline outlined in Table S3 below.

**Table S3: Schedule of Assessment for Implementation Outcomes for the CARIBOU-2 Intervention**

|  | **Month Since Initiation** | | | | | |
| --- | --- | --- | --- | --- | --- | --- |
|  | **-6** | **-3** | **0** | **12** | **24** | **52** |
| Phase I Fidelity | x | x |  |  |  |  |
| Phase II Fidelity |  | x | x |  |  |  |
| Phase III Fidelity |  |  |  | x | ------ | 🡪 |
| ICP Fidelity |  |  |  | x | ------ | 🡪 |
| Mood Foundations Fidelity |  |  |  | x | ------ | 🡪 |
| CBT Fidelity |  |  |  | x | ------ | 🡪 |
| BPI Fidelity |  |  |  | x | ------ | 🡪 |
| Caregiver Group Fidelity |  |  |  | x | ------ | 🡪 |
| Medication Algorithm Fidelity |  |  |  | x | ------ | 🡪 |
| Feasibility: Youth participant engagement checklist. |  |  |  | x | ------ | 🡪 |
| CFIR constructs: Qualitative Interview |  |  |  |  |  | x |

🡪 = until end of study period.
