# Supplementary material for: An Integrated Care Pathway for depression in adolescents: protocol for a Type 1 Hybrid Effectiveness-implementation, Non-randomized, Cluster Controlled Trial
Source: BMC Psychiatry. 2024 Mar 8;24:193. doi: 10.1186/s12888-023-05297-4 (PMC10921633; doi:10.1186/s12888-023-05297-4)
Supplement: Supplementary file 5 — Additional file 5: Appendix E. Informed consent to participate in a research study: youth version. [file 12888_2023_5297_MOESM5_ESM.docx]

**Appendix E: INFORMED CONSENT TO PARTICIPATE IN A RESEARCH STUDY: Youth Version**

**Study Title:** CARIBOU-2 Study: Effectiveness of an Integrated Care Pathway for Adolescent Depression

**[Site Representative and Information**: ________________________________]

**Funders:** This study is funded by Canadian Institutes for Health Research and Cundill Centre for Child and Youth Depression at CAMH

You are being asked to consider participating in a study. This study is a way of gathering information on treatments. This form explains the goal of this study, gives information about the study, the tasks involved, possible risks and benefits, and the rights of participants.

Please read this form carefully and ask any questions. You will have this form and all information concerning the study explained to you by a Research Analyst. You may take as much time as you wish to decide whether or not to participate. Feel free to discuss it with your family, or your therapist. Please ask the study team to clarify anything you do not understand or would like to know more about. Make sure all your questions are answered to your satisfaction before deciding whether to participate in this study. Participating in this study is your choice (voluntary). You have the right to choose not to participate, or to stop participating in this study at any time. Not participating or stopping participation will not affect your access to services at [site name].

**INTRODUCTION**

You are being invited to participate in a clinical trial (a type of study that involves research). You are eligible to participate in this trial because:

- You are between the ages of 13 to 18 (inclusive)
- You are expressing ‘depression” (or a similar synonym) as a concern
- The intake worker at **[site name]** or your therapist agrees that depressive symptoms are a concern
- Your Mood and Feelings Questionnaire (MFQ) score is ≥22 at two sequential visits (i.e., screening and baseline assessment)
- You are new to the site (in past 3 months) or have a period of no treatment for 3 months

**WHY IS THIS STUDY BEING DONE?**

The purpose of this study is to compare two models of treatment for depression in adolescents.

**WHAT WILL HAPPEN DURING THIS STUDY?**

If you decide to participate, you will be assigned to one of the two types of treatment. Your therapist can let you know which treatment you will be receiving. Both models could involve some combination of the following 7 components: Assessment, psychoeducation, psychotherapy, medication options, caregiver support, relapse prevention, team reviews, and measurement based care. You can receive the assigned treatment for up to 52 weeks and it will be delivered to you free of charge. Study participation is also up to 52 weeks. You do not have to be in the assigned treatment for 52 weeks in order to complete all study components. Participation can continue if you graduate/are discharged from the services at your site.

**HOW MANY PEOPLE WILL TAKE PART IN THIS STUDY?**

It is anticipated that about 300 youth will take part in this study, from research sites located in Ontario, Canada. This study will take at least eight years to complete. Results should be available by February 2029.

**WHAT ARE THE RESPONSIBILITIES OF STUDY PARTICIPANTS?**

If you decide to participate in this study you will be asked to do the following:

1. **Baseline Assessment:** Attend a virtual meeting, approximately 4hrs in length, to complete a diagnostic assessment. This meeting occurs via WebEx, a secure video chat platform. The purpose of the diagnostic assessment will be to describe you, your symptoms, and how you are doing. With your permission, it will be audio and video recorded for study staff training and inter rater reliability purposes. After coding and analyzing the information the audio and video recordings will be deleted. The information collected will be kept anonymous and any identifying information will be removed.
2. **Baseline Surveys:** You will be sent surveys you can complete on your own via REDCap. REDCap is a secure encrypted software for study data collection. It can take approximately 15mins to complete.
3. **Follow-up assessments:** You will be asked to attend additional virtual meetings, via Webex at the following time points: 4 weeks, 12 weeks, 24 weeks, 36 weeks and 52 weeks. During these meetings, you will be asked similar questions as in the baseline assessment; however, these meetings will be much shorter. They will range from 30mins to 90mins.
4. **Follow-up Surveys:** You will be sent surveys you can complete on your own via REDCap. These surveys will be similar to those completed at baseline. These surveys will be sent at the following time points: 2 weeks, 4 weeks, 12 weeks, 24 weeks, 36 weeks and 52 weeks. It will take anywhere from 5min to 20mins to complete.
5. **Services:** Engage in the treatment that you are receiving at your site. Treatment may or may not involve filling out surveys once a month that you will review with your therapist to further guide your treatment. These surveys can take approximately 15 minutes to complete, each time.
6. **Focus Groups:** A focus group is a small group of representative people who are asked to speak about their opinions. At the end of the 52 weeks, you may or may not be invited to discuss your experience of receiving treatment. With your permission, this focus group will be audio and video recorded. It will be conducted via WebEx. This focus group will take approximately 90mins to complete. This will be a group session, with other participants. You may be able to see other participants, and they may be able to see you during the focus group, if cameras are kept on. If you need to leave the group session, you will need to tell the group facilitator. It is important that you do not share the information that is discussed in the session with others (i.e., Do not share the meeting link with others or record the focus group meeting). Do not invite others to join you on the call. There is a small risk that somebody may be recording or will share information from the call, even though everyone will be told not to. When you enter your display name in Webex, the name you enter will be visible to others in the group. If you want, you can use your first name only, initials, a made-up name, or the study team can provide you with a code number.
7. **Chart review**: Your therapist is required to keep notes about the sessions that they have with you, while providing care. By signing this consent form, you are providing the study team with permission to access these notes. The study team will only access relevant information, for chart review purposes. Your therapist will be aware that you are participating in the study.

**WHAT ARE THE RISKS OR HARMS OF PARTICIPATING IN THIS STUDY?**

We do not expect much discomfort from participating in this study. However, answering the assessment questions and surveys may cause some discomfort because the questions are personal. If you feel discomfort and wish to discuss it, the Research Analyst will provide you with a Resource Card, which outlines additional services and support that you can access. You also have full autonomy on the information you would like to provide for this study. This means you can skip/pass any question you do not want to answer without having to provide a reason. Teleconferencing/videoconferencing will be done using Webex, a secure platform used by CAMH. Like online shopping, teleconferencing/videoconferencing technology has some privacy and security risks. It is possible that information could be intercepted by unauthorized people (hacked) or otherwise shared by accident. This risk cannot be completely eliminated. We want to make you aware of this.

**WHAT ARE THE BENEFITS OF PARTICIPATING IN THIS STUDY?**

By participating in this study, you will be helping researchers by giving a youth’s perspective. Your participation will also benefit other clients by helping to shape the services offered to future youth and their families.

**CAN PARTICIPATION IN THIS STUDY END EARLY?**

You can choose to end your participation (i.e., withdrawal) at any time. If you choose to withdraw, you are encouraged to tell your therapist and the study team. The study team can be reached at [**caribou2.study@camh.ca**](mailto:caribou2.study@camh.ca)

There are two types of withdrawal. You can choose not to continue in the study, but allow previously collected information to still be used in the study. Or you can choose not to continue in the study and have all collected information removed from the study. As previously mentioned, withdrawing from the study will not affect you access to services at your site.

Your therapist and/or the study team can also stop your participation in the study early, and without your consent, for reasons such as:

- You are unable to tolerate the treatment
- New information shows that the treatment is no longer in your best interest
- The therapist no longer feels this is the best option for you
- **[name of site]** or CAMH decides to stop the study
- The Research Ethics Board at **[site name]** or CAMH withdraw permission for this study to continue

If this happens, it may mean that you would not receive an intervention for the full period described in this consent form. If you are removed from the study, your therapist will discuss the reasons with you and plans will be made for your continued care at **[site name]** outside of the study.

**WHAT HAPPENS IF I HAVE A RESEARCH RELATED INJURY?**

In the case of research-related side effects or injury, medical care will be provided by your doctor or you will be referred for appropriate medical care OR in the same way you would normally get medical care (for example, by going to your family doctor or seeking emergency medical treatment if needed). The costs of your medical treatment will be paid for by the provincial medical plan or by seeking reimbursement from your private medical insurer (if any) to the extent that such coverage is available. There may be extra costs that are not covered by provincial insurance or your private medical plan. Examples of these extra costs could be medications or other needed medical treatments to treat side effects that you may experience. If you have private health care insurance, the insurer may not pay for these added costs.

**IS THERE COMPENSATION FOR PARTICIPATING?**

At the first and last research meeting, you will receive a $50 e-gift card, of your choosing from the selection made available. At each of the other completed research meeting time points, you will receive a $25 e-gift card, of your choosing from the selection made available. You can also receive volunteer hours towards school credit.

**HOW WILL MY INFORMATION BE KEPT CONFIDENTIAL?**

You have the right to have any information about you and your health that is collected, used or disclosed for this study to be handled in a confidential manner. If you participate in this study, the investigator(s) and study team will look at your personal health information and collect only the information they need for this study. “Personal health information” is health information about you that could identify you because it includes information such as your:

- Name
- Address,
- Telephone number
- Date of birth
- New and existing medical records
- The types, dates and results of various tests, treatments, and procedures

You have the right to access, review and request changes to your personal health information. Access to your personal health information will take place under the supervision of the Principal Investigator. As per the policy at the Centre for Addiction and Mental Health, the principal investigator will keep any personal health information about you in a secure and confidential location for 10 years..

“Study data" is health information about you that is collected for the study, but that does not directly identify you. Any study data about you will have a Study ID and will not contain your name or address, or any information that directly identifies you. Study data may be shared and used by other study teams for other studies (i.e., secondary analyses studies). The investigator(s), study team and the other individuals (i.e., REB members) will keep the information they see or receive about you confidential, to the extent permitted by applicable laws. Even though the risk of identifying you from the study data is very small, it can never be completely eliminated.

When the results of this study are published, your identity will not be disclosed. You have the right to be informed of the results of this study once the study is complete. If you would like to be informed of the results of this study, please provide your name, address and telephone number to the Research Analyst assigned to this study. A description of this clinical trial will be available on <http://www.ClinicalTrials.gov>. This website will not include information that can identify you. At most, the website will include a summary of the results. You can search this website at any time.

**ARE THERE ANY CONFLICTS OF INTEREST/RELATIONSHIPS?**

[Site Name] may be receiving financial payment from the Centre for Addictions and Mental Health to cover costs associated with this study.

**COMMUNICATION WITH YOUR FAMILY DOCTOR**

Your family doctor may be informed that you are taking part in this study so that your therapist and family doctor can help you make informed decisions about your medical care. If you do not want your family doctor/health care provider to be informed, please discuss this with your therapist.

**WHAT ARE THE RIGHTS OF PARTICIPANTS IN A RESEARCH STUDY?**

You have the right to receive all information that could help you make a decision about participating in this study. You also have the right to ask questions about this study and your rights as a research participant, and to have them answered to your satisfaction, before you make any decision. You also have the right to ask questions and to receive answers throughout this study.

**WHOM DO PARTICIPANTS CONTACT FOR QUESTIONS?**

If you have questions about taking part in this study, you can reach out to the CARIBOU-2 study team by email at [**caribou2.study@camh.ca**](mailto:caribou2.study@camh.ca)

The CAMH Research Ethics Board has reviewed and approved this study. If you have questions about your rights as a study participant or any ethical issues related to this study that you wish to discuss with someone not directly involved with the study, you may call the **Chair of the CAMH Research Ethics Board at** 416-535-8501 ext. 36798**.**

**Study Title:** CARIBOU-2 Study: Effectiveness of an Integrated Care Pathway for Adolescent Depression

**DOCUMENTATION OF INFORMED CONSENT**

You will be given a fully executed copy of this informed consent form.

By signing this form, I confirm that:

- This study has been fully explained to me and all questions were answered to my satisfaction
- I understand the requirements of participating in this study
- I have been informed of the risks and benefits, if any, of participating in this study
- I have been informed of any alternatives to participating in this study
- I have been informed of the rights of research participants
- I authorize access to and use of my personal health information, medical records, and study data as explained in this form
- I understand that my therapist and/or family doctor may be informed of my participation in this study

_______________________ ____________________ _____________________

Name of participant (print) Signature Date

Person obtaining consent

By signing this form, I confirm that:

- This study and its purpose has been explained to the participant named above
- All questions asked by the participant have been answered
- A copy of this signed and dated document to the participant

_______________________ ____________________________ _____________________

Name of Person obtaining Signature Date

consent (print)

_____________________ _____________________ _____________________

Name of Witness (print) Signature Date
