# Supplementary material for: An Integrated Care Pathway for depression in adolescents: protocol for a Type 1 Hybrid Effectiveness-implementation, Non-randomized, Cluster Controlled Trial
Source: BMC Psychiatry. 2024 Mar 8;24:193. doi: 10.1186/s12888-023-05297-4 (PMC10921633; doi:10.1186/s12888-023-05297-4)
Supplement: Supplementary file 6 — Additional file 6: Appendix F. The Data Monitoring Committee Charter. [file 12888_2023_5297_MOESM6_ESM.pdf]

Appendix F: The Data Monitoring Committee Charter

Title of Protocol: Effectiveness of an Integrated Care Pathway for Adolescent Depression: Protocol for a Multi-site Stepped-Wedge, Cluster-Randomized Controlled Trial

Protocol Number: 019/2021

Sponsor of Protocol: Centre for Addictions and Mental Health

Date of Document: January 18th 2022

1. INTRODUCTION

2. PRIMARY RESPONSIBILITIES
   a. Responsibilities of the Data Monitoring Committee 3
   b. Responsibilities of the Sponsor 3
   c. Responsibilities of the Data Coordinating Centre 3
   d. Responsibilities of the Statistical Data Analyst Centre 3

3. ORGANIZATIONAL DIAGRAM 4

4. MEMBERSHIP OF THE DATA MONITORING COMMITTEE
   a. Members 4
   b. Conflicts of Interest
   c. Indemnity 5

5. TIMING AND PURPOSE OF THE DMC MEETINGS
   a. Organizational Meeting 5
   b. Early Safety/Trial Integrity Reviews 5
   c. Formal Interim Analysis Meeting 5
   d. Unscheduled Meetings 5

6. PROCEDURES TO ENSURE CONFIDENTIALITY AND PROPOER COMMUNICATION
   a. Closed Sessions 5
   b. Open Sessions 5
   c. Minutes of the DMC Meetings 6
   d. Recommendations to the Sponsor and/or Steering Committee 6

7. STATISTICAL MONITORING GUIDELINES 6

8. CONSENT OF THE DMC'S OPEN AND CLOSED REPORTS
   a. Open Statistical Report: An Outline 6
   b. Closed Statistical Report: An Outline 6

9. DMC APPROVAL AND SIGNATURES 7

1. INTRODUCTION

This Charter is for the Data Monitoring Committee (DMC) for the current study called, "Effectiveness of an Integrated Care Pathway for Adolescent Depression: Protocol for a Multi-site Stepped-Wedge, Cluster-Randomized Controlled Trial". This Charter defines the primary responsibilities of the DMC, its relationship with the RCT components, and the purpose and timing of meetings. It also provides procedures (i.e., ensuring confidentiality, statistical monitoring guidelines, and reports).

2. RESPONSIBILITIES

a) Responsibilities of the Data Monitoring Committee

The Data Monitoring Committee (DMC) will be responsible for:

1. Safeguarding the interests and safety of current and future study participants
2. Preserving integrity and credibility of the trial in order that future patients are treated optimally
3. Identifying Severe Adverse Events (i.e., death by any cause, suicide attempt, hospitalization for mental health reasons and potential link to intervention)

b) Responsibilities of the Sponsor

CAMH is the sponsor for this RCT; responsibilities include:

- Conduct all financial procedures, as the grant holder
- Provide all required reporting to funder (CIHR)
- Maintain the integrity and reliability of all of the data
- Provide materials and resources to the DMC when necessary
- Promptly informing the DMC of any safety concerns throughout the RCT
- Communicating DMC recommendation in writing to Principal Investigators and Co-Principal Investigators
- Expediting reporting of adverse events

c) Responsibilities of the Data Coordinating Centre

CAMH is also the Data Coordinating Center; responsibilities include:

- Collection and onsite monitoring of case reports
- Collection, monitoring, and management of all data collected
- Providing analysis data sets to the DMC when requested

d) Responsibilities of the Statistical Data Analysis Centre: CAMH is also the Statistical Data Analysis Centre. Dr. Wei Wang is the primary statistical programmer, who will be responsible for the overall data analysis and generation of statistical reports requested by the DMC. Requested reports may include: interim results on primary and secondary clinical outcomes, and emerging

data on adverse events. Dr. Claire De Oliveira will be responsible for providing any data and findings related to the ICP implementation.

3. ORGANIZATIONAL DIAGRAM

Site selection has been completed.

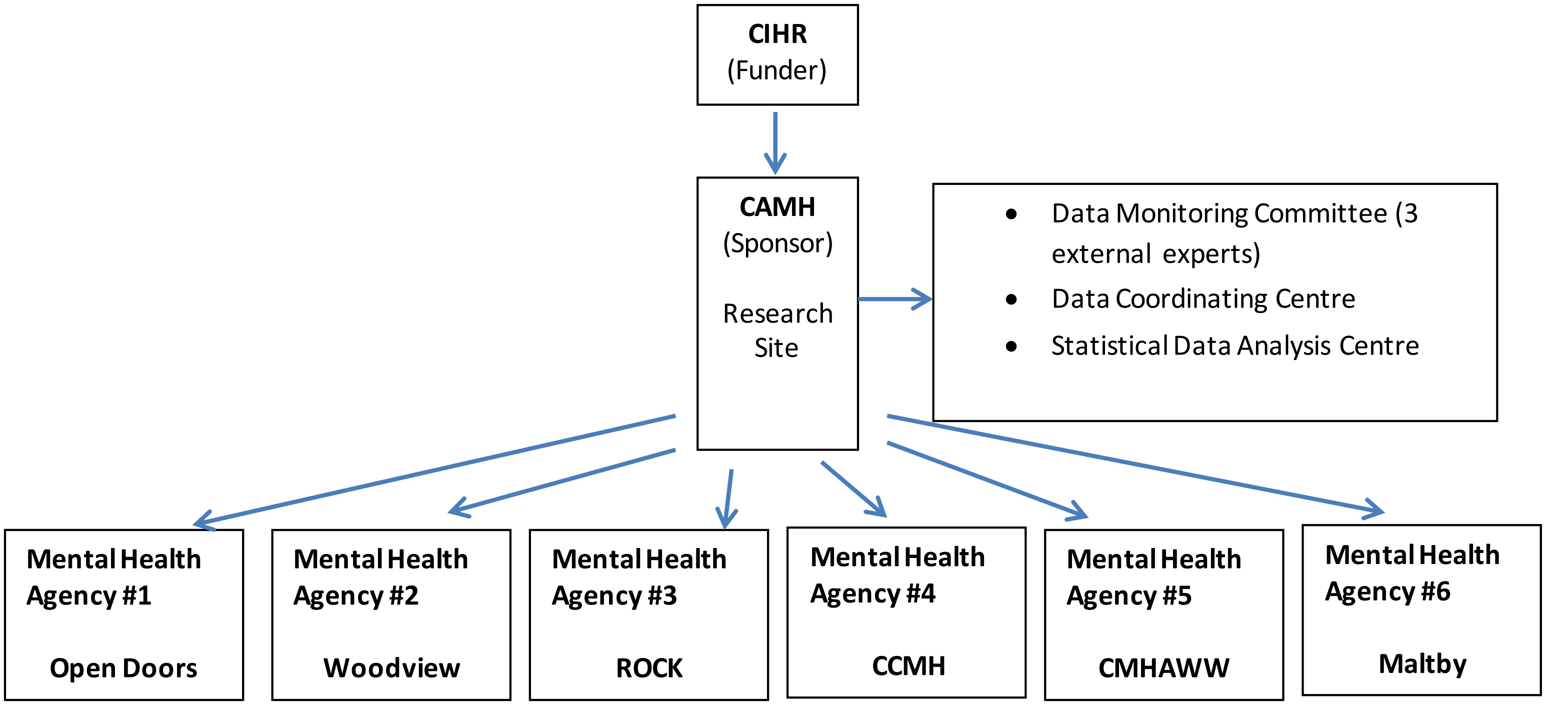

4. MEMBERSHIP OF THE DATA MONITORING COMMITTEE

Members: The DMC consists of three individuals external to the CARIBOU-2 project.

1) Dr. Lehana Thabane, PhD, Professor and Associate Chair, Health Research Methods, Evidence, and Impact, University of McMaster. Chair of the DMC.

2) Dr. Suneeta Monga, MD, Associate Psychiatrist-in-Chief, Department of Psychiatry at the Hospital for Sick Children. Associate Professor, Department of Psychiatry at the University of Toronto. DMC member.

3) Dr. Khrista Boylan, PhD, Associate Professor, University of McMaster. DMC member.

Conflicts of Interest: The DMC does not include individuals that are principal investigators, co-principal investigators, or co-investigators for this RCT. It also does not include individuals that will be recruiting and managing the trial and its participants. Therefore, the DMC consists of individual that are not affiliated with the RCT.

Indemnity: DSMB members are covered under the sponsor's policy—the Healthcare Insurance Reciprocal of Canada (HIROC) policy—as long as members are doing work for or on behalf of CAMH. Any liability arising out of the work of the DMC and its members will be covered by the HIROC policy; however, is subject to the facts and circumstances of a claim and the terms and conditions of the policy.

5. TIMING AND PURPOSE OF THE DMC MEETINGS

DMC meetings will only occur when necessary (i.e., when adverse events occur). All meetings will be done via WebEx and will be between 30mins-60mins in duration. Meetings will include the DMC and required members (i.e., Principal Investigator and Research Coordinator).

Organizational Meeting: The initial meeting of the DMC is scheduled for March 1st 2021 at 11:00am. It will be held after the protocol and other documents (i.e., TAHSN) has been finalized and submitted to the Research Ethics Board for review. The purpose of this meeting is to provide a brief overview of the RCT and coordinate future DMC meetings, topics of discussions, and tasks. The DMC will receive the protocol prior to this meeting and may discuss any concerns or questions they may have about the RCT. **Unscheduled Meetings:** Unscheduled meetings are not anticipated. At a schedule meeting, the DMC will determine the next meeting or whether an additional monthly meeting is necessary.

6. PROCEDURES TO ENSURE CONFIDENTIALITY AND PROPOER COMMUNICATION

To ensure the integrity and credibility of the RCT, procedures will be created and implemented to ensure that the DMC have access to the required information (i.e., aggregated clinical data). Requests will be made to the Research Coordinator. The Research Coordinator will work with the Statistical Data Analysis Centre to ensure the required data is accurate and appropriate to share (i.e., data has been properly de-identified).

a) **Closed Sessions:** Closed sessions involve only the DMC and Statistical Data Analysis Centre personnel (i.e., Dr. Wang). During this meeting, the DMC will discuss confidential data from the RCT, including information about the relative efficacy and safety of the treatment (known as CARIBOU-2). The DMC will develop consensus on a list of recommendations (if necessary) that relate to the continuation of the RCT.

b) **Open Sessions:** Open sessions will include the DMC, Statistical Data Analysis Centre personnel, study investigators (Dr. Darren Courtney and Dr. Melanie Barwick), and the Research Coordinator (Michelle Ferreira). These meetings are known as the monthly meetings and will be used to discuss the RCT and deliverables progress, challenges, adaptations, and concerns.

c) **Minutes of the DMC Meetings**: Prior to each monthly meeting, the Research Coordinator will provide the DMC with an agenda—the topics to be discussed during the meeting. Meeting minutes will be taken by the Research Coordinator during the meeting, saved on a secure drive (X Drive) and also sent to the DMC.

d) **Recommendations to the Sponsor and/or Steering Committee:** Formal recommendations by the DMC will be written and sent to the respective parties (i.e., Principal Investigator and Steering Committee). Recommendations can be reviewed and discussed at the next DMC meeting.

7. STATISTICAL MONITORING GUIDELINES

The statistical monitoring guidelines are still in development.

8. CONSENT OF THE DMC'S OPEN AND CLOSED REPORTS

a) **Outline of an Open Statistical Report:** Open Statistical Reports may include the following:
- One page summary of the study design
- Statistical commentary of insights that include figures and tables, matching the concerns identified
- DMC recommendations made prior to a meeting
- Any major study changes (i.e., to the protocol)
- Information regarding participant screening
- Study accrual by month at each site (if necessary)
- Baseline participant characteristics (i.e., demographics)
- Study violations or overrides (i.e., participation eligibility)
- Adherence to project timeline
- Study status at each site (i.e., treatment discontinuations, study withdrawals—site and participant level, participant retention)

b) **Outline of a Closed Statistical Report:** Closed Statistical Reports may include the following:
- Statistical commentary on insights during closed meetings, which include figures and tables related to concerns or issues DMC monitoring plan and summary of closed report data presented during closed meetings
